# Fungal diversities and community assembly processes show different biogeographical patterns in forest and grassland soil ecosystems

**DOI:** 10.3389/fmicb.2023.1036905

**Published:** 2023-02-01

**Authors:** Min Wang, Can Wang, Zhijun Yu, Hui Wang, Changhao Wu, Abolfazl Masoudi, Jingze Liu

**Affiliations:** Hebei Key Laboratory of Animal Physiology, Biochemistry and Molecular Biology, Hebei Collaborative Innovation Center for Eco-Environment, Hebei Research Center of the Basic Discipline of Cellular Biology, Ministry of Education Key Laboratory of Molecular and Cellular Biology, College of Life Sciences, Hebei Normal University, Shijiazhuang, Hebei, China

**Keywords:** soil fungal community, geographical gradient, distribution pattern, community assembly, trophic mode

## Abstract

Soil fungal community has been largely explored by comparing their natural diversity. However, there is a relatively small body of literature concerned with fungal community assembly processes and their co-occurrence network correlations carried out across large spatial–temporal scales with complex environmental gradients in natural ecosystems and different habitats in China. Thus, soil fungal community assembly processes were assessed to predict changes in soil function in 98 different forest and grassland sites from the Sichuan, Hubei, and Hebei Provinces of China using high-throughput sequencing of nuclear ribosomal internal transcribed spacer 2 (ITS-2). The 10 most abundant fungal phyla results showed that Ascomycota was the most abundant phylum in forests from Sichuan province (64.42%) and grassland habitats from Hebei province (53.46%). Moreover, core fungal taxa (487 OTUs) represented 0.35% of total fungal OTUs. We observed higher fungal Shannon diversity and richness (the Chao1 index) from diverse mixed forests of the Sichuan and Hubei Provinces than the mono-cultured forest and grassland habitats in Hebei Province. Although fungal alpha and beta diversities exhibited different biogeographical patterns, the fungal assembly pattern was mostly driven by dispersal limitation than selection in different habitats. Fungal co-occurrence analyses showed that the network was more intense at Saihanba National Forest Park (SNFP, Hebei). In contrast, the co-occurrence network was more complex at boundaries between forests and grasslands at SNFP. Additionally, the highest number of positive (co-presence or co-operative) correlations of fungal genera were inferred from grassland habitat, which led fungal communities to form commensalism relationships compared to forest areas with having higher negative correlations (mutual exclusion or competitive). The generalized additive model (GAM) analysis showed that the association of fungal Shannon diversity and richness indices with geographical coordinates did not follow a general pattern; instead, the fluctuation of these indices was restricted to local geographical coordinates at each sampling location. These results indicated the existence of a site effect on the diversity of fungal communities across our sampling sites. Our observation suggested that higher fungal diversity and richness of fungal taxa in a particular habitat are not necessarily associated with more complex networks.

## Introduction

Soils are considered the most diverse microbial habitat, and soil biota with great diversity play a highly significant role in soil health (Sutcliffe et al., 2018). In the soil ecosystem, fungi (domain Eukarya) functioning as pathogens, decomposers, and mutualists, reportedly leads to the alteration of most soil nutrients (Buée et al., 2009; Sutcliffe et al., 2018). In addition, fungi play an essential role in nearly all soil biogeochemical cycles (Gadd, 2007). The diversity of the fungal communities in soils is heavily regulated by a variety of biotic and abiotic ecological variables, including temperature, moisture, seasons, soil edaphic parameters, plant and vegetation types, geographical coordinates, and even agricultural practices (Singh et al., 2009; Lange et al., 2014; Shi et al., 2014; Ranelli et al., 2015; Thomson et al., 2015; Thakur and Geisen, 2019). Thus, specifying the mechanisms underlying fungal biogeographic patterns can provide perspicuity into how biodiversity is developed and sustained (Thakur et al., 2020). The existing literature on biogeographic patterns of fungal diversity is extensive and focuses ranged from local to global scale over the past few decades (Tedersoo et al., 2012; Zhou et al., 2016; Bowman and Arnold, 2021), and it has been highlighted that the community homogeneity between fungal taxa declines with increasing geographical distance (Soininen et al., 2007; Xiao et al., 2018). Recent studies have accentuated the vital role of regional fungal species diversity and community assembly processes in driving fungal biogeography (Myers et al., 2015; Zhang et al., 2020). Yet, few studies have examined ecological community patterns of fungal diversity at multiple spatial scales with varying environmental gradients, especially in different ecosystems, considering local community assembly processes and regional fungal diversity in China (Shi et al., 2014; Liu et al., 2021; Zheng et al., 2021).

In recent years, an increasing number of studies have used the high-throughput sequencing approach (HTS) based on the ITS region to evaluate the diversity of fungal communities in forest habitats as well as grasslands (Talbot et al., 2014; Yang et al., 2019; Chen et al., 2021). Naturally, forest regions grow in multiple layers (Baldrian, 2016) in comparison with that of most grassland habitats with less dense vegetation, indicating the high spatial heterogeneity of multi-layered vegetation of forests may potentially lead to the high degree of diversity and richness seen in fungal community compositions (Erdős et al., 2019). Thus, it is expected that fungal communities in forest ecosystems should be more complex and intense. These studies mainly focused on fungal community diversity by comparing their natural diversity and distribution and less applied the assemblage of fungal community and their co-occurrence network correlations (Shi et al., 2020). There is still a lack of discussion and analysis based on the fungal co-occurrence network on the whole fungal communities across large spatial–temporal scales in natural ecosystems and different habitats in China.

The response of ecosystems to environmental changes can ultimately influence ecological processes that control the assembly of soil microbial communities, which can result in substantial deviation in β diversity (higher community composition) when the environmental conditions are heterogenous and/or vice versa (Liu et al., 2021). Two sorts of processes, deterministic (niche-based) and stochastic (neutral), are mainly responsible for impacting soil microbial processes (Stegen et al., 2012; Huang et al., 2021). It has been expected that deterministic processes, including environmental filters, biotic interactions (antagonistic and synergistic), and species traits, are the foundation for the niche-based theory to motivate the local community composition toward a steady phase (Chesson, 2000). In contrast, stochastic processes, including dispersal, ecological drift, and stochastic diversification, are the basis for neutral theory and exhibit random changes in microbial communities (Hubbell, 2005) that result in patterns of community composition indistinguishable from random assemblages (Chave, 2004). The relative contribution of these two main microbial assembly processes can be defined by several statistical analyses such as neutral models, null models, and multivariate analysis (Chesson, 2000; Hubbell, 2005; Stegen et al., 2012; Huang et al., 2021; Hussain et al., 2021; Liu et al., 2021). However, the relevant importance of the two processes remains less understood for fungal community assembly processes in different habitats in China.

In this study, we hypothesized that: (H_1_) due to forest regions’ growth in multiple layers in comparison with grassland habitats, the high interaction of fungal communities in forest ecosystems would be expected rather than in grassland habitats. The dependencies and interactions of fungal taxa were inferred by identifying the number of positive (co-presence) and negative (mutual exclusion) correlations; (H_2_) a clearly distinct community assembly pattern would be observed for the fungal community assembly processes in different habitats. To understand further the relevant importance of the fungal community assembly processes in different habitats in China, the present study investigated the natural distribution of whole fungal operational taxonomic units (OTUs) compositions and fungal community assembly processes of soils in Sichuan, Hubei, and Hebei Province of China using the deep soil high-throughput sequencing approach. Additionally, the influence of geographical coordinates was individually evaluated for each sampling location on fungal diversity using a generalized additive model (GAM).

## Materials and methods

### Study sites

**Figure 1:**
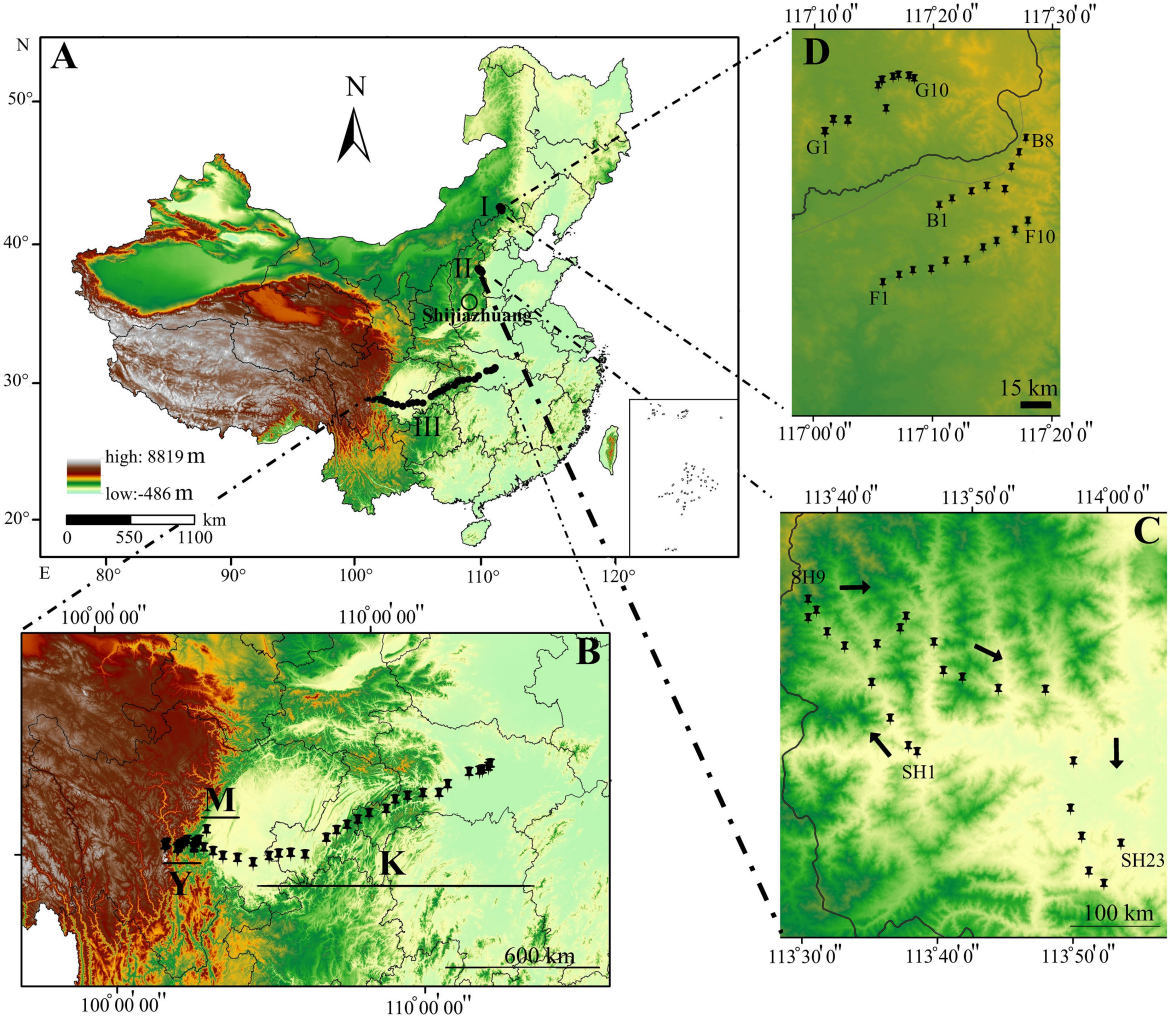
Map of China based on the elevational pattern **(A)**. Sampling sites from Saihanba National Forest Park (SNFP) **(B)**. Sampling sites on the Taihang Mountains **(C)**. Sampling sites from Kangding: 29°49′516″N, 102°54′276″E, elevation ~3,100 m above sea level) and central Sichuan Province to southeastern Hubei Province (Ping Ke Xian: 31°06′962″N, 113°15′18″E, elevation 100 m above sea level) **(D)**. The black dots indicate the sampling path from Sichuan Province to Hubei Province. The geographical map with coordinates was created by ArcGIS for Windows 10.2 (Environmental Systems Research Institute (ESRI; www.esri.com; 2012). B indicates boundaries between forest and grassland at SNFP; G, grasslands at SNFP; F, forests at SNFP; SH, the Taihang Mountains; K, Meishan to Ping Ke Xian; Y, Ya’an area; and M, Meishan region.

Our sampling was conducted in the area scattered across a gradient stretching from west to east in southwestern China, situated in both Sichuan and Hubei provinces. Soil collections were also conducted from Hebei Province’s Taihang Mountains and Saihanba National Forest Park from July to August 2018 (Figure 1). In total, 98 sites were surveyed, and 490 soil samples were collected (Figure 1A). Five lines were randomly selected at each sampling site, with a distance of 100 m between each pair. Five sub-soil samples were collected from each line with an approximate length of 10 m between two microsites of each line. These five soil slices were combined to form a single composite sample (Masoudi et al., 2018). Our investigation focused on three regions in China, from the south (Sichuan and Hubei) and north (Hebei province), with large biogeographical scales. The first sampling forest area stretched an 1,350 km from southwestern (Kangding: 29°49′516″N, 102°54′276″E, elevation ~3,100 m above sea level) and central Sichuan Province to southeastern Hubei Province (Ping Ke Xian: 31°06′962″N, 113°15′18″E, elevation 100 m above sea level; Figure 1B). In this area, sampling was conducted at 47 sampling sites by collecting 14 soil samples from Kangding to Ya′an (hereafter abbreviated as Y; Y1-Y14), seven soil samples from the Meishan region (hereafter abbreviated as M; M1-M7), and 26 soil samples from Meishan to Ping Ke Xian (hereafter abbreviated as K; K1-K26). The climate of this region can be classified as wet subtropical, and the vegetation type is characterized mainly as tropical-subtropical plants. Additionally, some plant genera such as *Abies*, *Picea*, *Pinus*, *Tsuga*, *Larix*, and *Sabina* are widely distributed. The annual temperature in this region ranges from 6°C to 10°C, whereas the yearly precipitation averages 69–800 mm. This region lies on the border between subtropical and tropical; therefore, its landscape is notably diverse. The distance between sampling sites at Y and M sampling locations was ~10 km, whereas the distance between the sampling sites at location K was nearly 50 km (~1,350 km). The second soil sampling site was at the Taihang Mountains, located west of Hebei Province, and was conducted at 23 different sites, with ~10 km between each pair of sampling sites (Figure 1C). The Taihang Mountains, which extend from 34°14′N to 41°6′N and from 110°13′E to 116°34′E, and are close to Shijiazhuang (SH; SH1-SH23), the capital city of Hebei Province, exhibit a warm temperate and semi-humid continental monsoon climate, with a mean annual temperature ranging from −9.1°C to 21.6°C and yearly precipitation ranging from 318 to 817 mm (Zhang et al., 2006). The area is mostly covered by grassland, shrub, cropland, and broadleaved forest. Some plant species, such as *Hippophae rhamnoides*, *Vitex negundo* var. *heterophylla*, *Corylus heterophylla*, *Spirea trilobata* of shrub communities, *Betula platyphylla*, and *Quercus liaotungensis* of forest communities, are considered the dominant vegetation types in this region (Li et al., 2013; Hu et al., 2019). The third soil sampling site was at the Saihanba National Forest Park (SNFP, including planted forest and grassland habitats) in Weichang County in Chengde, Hebei Province, which is China’s largest plantation forest (94,700 ha; Deng et al., 2016; Figure 1D). The average annual precipitation and temperature of SNFP are ~450 mm and −1.4°C, respectively (Yang et al., 2014). Additionally, most precipitation occurs from June to August (Ma et al., 2013). Soil sampling at SNFP with a semi-natural forest mosaic from Hebei Province was performed in three different vegetation biome types as follows: forest represented by 10 samples (F; F1-F10), grassland represented by 10 samples (G; G1-G10), and boundaries between grassland and forest habitats represented by eight samples (B; B1-B8; Supplementary Table S1).

### Soil DNA extraction, PCR amplification, and ion torrent S5™ XL sequencing

DNA extraction was performed by using DNeasy^®^ PowerSoil^®^ Kit (QIAGEN, Hilden, Germany) to obtain genomic DNA free of PCR-inhibitory phenolic compounds following the manufacturer’s instructions. The two DNA extractions per soil sample were pooled before further analysis. Primers ITS3-2024F (or ITS3: 5′-GCATCGATGAAGAACGCAGC-3′) and ITS4-2409R (or ITS4: 5′-TCCTCCGCTTATTGATATGC-3′) were used to amplify 350 bp of the fungal rRNA gene internal transcribed spacer-2 (ITS-2) region (White et al., 1990; Bellemain et al., 2010). This primer pair contains an 8-nucleotide barcode sequence unique to the fungal ITS2 region (Wu et al., 2021). Each PCR reaction was in a final volume of a 30 μL and contained: 15.0 μL of Phusion^®^ High-Fidelity PCR Master Mix (New England Biolabs, Ipswich, MA, United States) with HF Buffer (New England Biolabs), 7.5 μL of ddH_2_O, 5.0 μL of the DNA sample, 2.0 μL of forward and reverse primers (10 mM), and 0.5 μL of bovine serum albumin (TaKaRa, Tokyo, Japan). PCR cycling conditions were: 98°C for 10 s as initial denaturation, 30 cycles of 98°C for 10 s, 50°C for 30 s, and 72°C for 30 s, and a 5 min incubation at 72°C. The PCR products were purified using GeneJET™ Gel Extraction Kit (Thermo Fisher Scientific, Waltman, MA, United States) and quantified by Nanodrop 2000 (Thermo Fisher Scientific). Then, the prepared libraries were sequenced on an Ion S5™ XL SE400/SE600 next-generation sequencing system (Thermo Fisher Scientific, Waltham, MA, United States).

### Bioinformatic and statistical analyses

According to our previous publications, obtained data were analyzed (Masoudi et al., 2020; Wang et al., 2020, 2021; Yang et al., 2022). Basic information was collected during the construction of OTUs, such as valid tags data, low-frequency tags data, and annotation data of tags (Supplementary Figure S1; Supplementary Table S2; Supplementary Information). Paired-end (PE) reads were merged into a sequence, and the minimum overlap length was 10 bp using FLASH ver. 1.2.7.^1^ The obtained raw tags were analyzed with QIIIME (Quantitative Insights Into Microbial Ecology ver. 1.9.1; Caporaso et al., 2010) and Cutadapter ver. 1.9.0 (Martin, 2011) with manipulated settings to check filtering quality. Details of all analyses are provided in the Supplementary Information. All sequence data were previously deposited in the National Center for Bioinformatics Information (NCBI) Sequence Read Archive database under the BioProject ID PRJNA551928, Submission ID SUB5873947.

## Results

### Composition of the soil fungal community and analysis of the reads by OTU clustering

**Figure 2:**
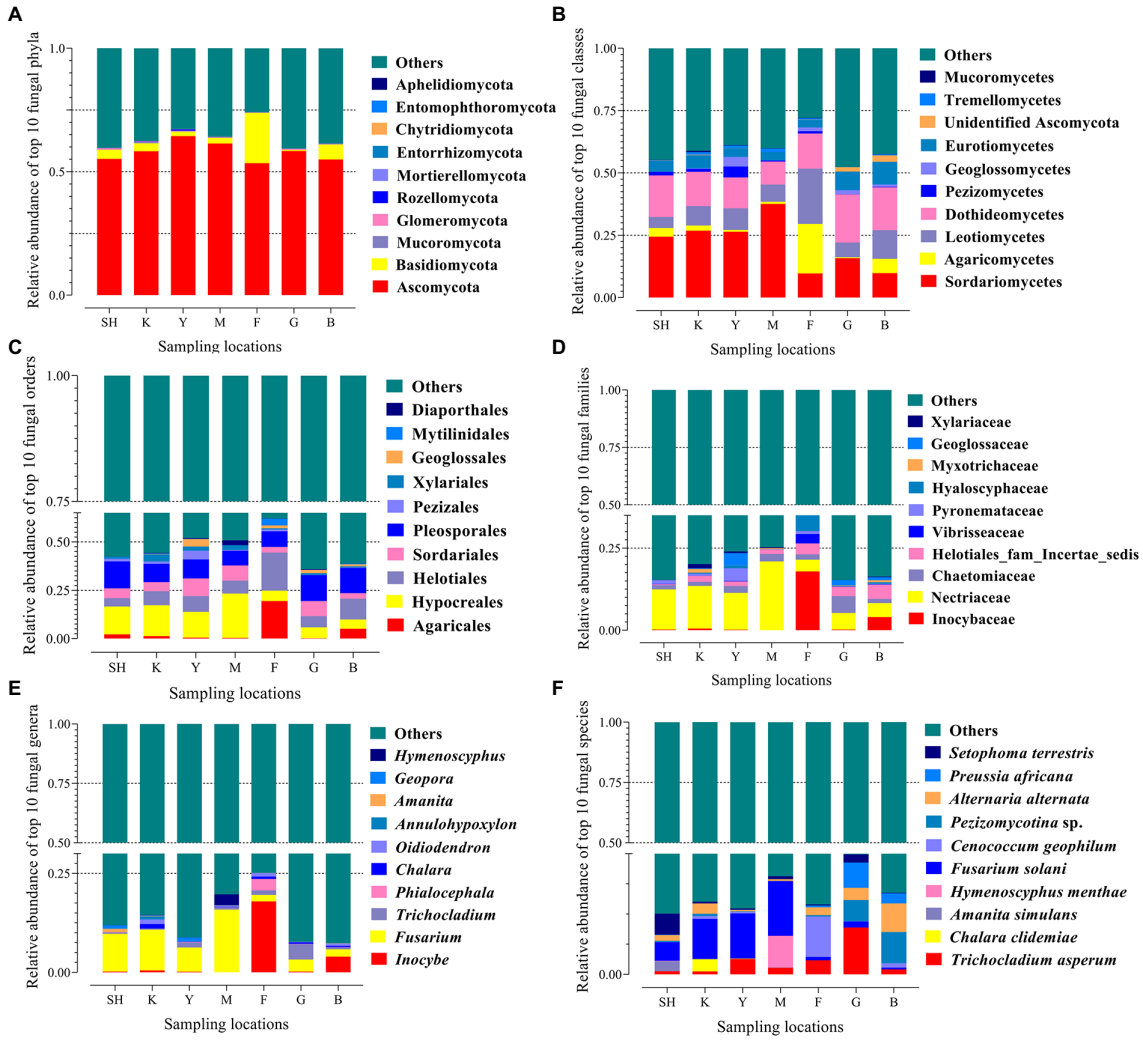
Relative abundance of the dominant fungal taxa in seven sampling locations from Sichuan, Hubei, and Hebei Provinces in China. Each bar represents the top ten fungal OTUs at the phylum level **(A)**, class **(B)**, order **(C)**, family **(D)**, genus **(E)**, and species **(F)** ranked by the relative abundance in each sampling location, and different colors represent the frequency of the different OTUs. The designation of ‘other’ indicates that those sequences were not classified into any known group. Abbreviations of variables are given in Figure 1.

A total of 7,497,683 ITS-2 sequences were obtained. Having removed low-quality and chimeric sequences, the average number of valid reads generated per sample was found to be 61,018, with a median read length of 305 bp. Species annotations were made *via* comparison of 11,593 OTUs with the database UNITE, based on a minimum identity of 97%, resulting in a classification of 10,132 OTUs (87.40%). Accordingly, annotated sequences that were classified as a kingdom, phylum, class, order, family, genus, and species corresponded to 87.40%, 45.91%, 42.55%, 38.81%, 29.27%, 24.60%, and 12.58% of total taxa, respectively. The number of valid reads per sample ranged from 54,011 (SH13) to 91,082 (G8). The number of OTUs per sample ranged from 867 (SH1) to 1,528 (SH6), where 1,403 OTUs were calculated on average. On average, the lowest number of OTUs was recorded for data from the G collection site, whereas the highest OTU numbers were recorded for those from the Y collection site (Supplementary Table S1). Unclassified (no BLAST hit) groups were considered unknown fungal taxa and not assigned to any known group. The number of OTUs increased with the number of reads, and a plot of OTUs versus the number of ITS-2 sequences yielded rarefaction curves that approached a plateau (Supplementary Figure S2). The rarefaction curves showed that the samples were grouped in two levels according to the climate zone. The rarefaction curves showed species accumulation in the cold temperate region (SNFP) and the Taihang Mountains were more moderate than in K and Y sampling locations (Supplementary Figure S2). The result of the species accumulation plot showed a satisfactory representation of the most common fungal taxa of these communities harbored, indicating the effectiveness of the sampling size (Supplementary Figure S3). Clean sequences were clustered in order to find the most abundant fungal taxa in the soil samples (Marčiulynas et al., 2022). The ten most abundant fungal taxa from phylum to species level are shown (Figure 2). Ascomycota was the most abundant phylum in forest and grassland habitats, with the highest (64.42%) and lowest (53.46%) abundance levels at Y and F sampling sites, respectively. The other dominant fungal species were found in fungal phyla such as Basidiomycota, Mucoromycota, Glomeromycota, Rozellomycota, Mortierellomycota, Entorrhizomycota, Chytridiomycota, Entomophthoromycota, and Aphelidiomycota (Figure 2A). The most abundant fungal classes detected were Sordariomycetes (highest frequency at site M: 37.56%), Agaricomycetes (highest frequency at site F: 19.86%), Leotiomycetes (highest frequency at site F: 22.18%), and Dothideomycetes (highest frequency at site G: 19.32%; Figure 2B). At the order level, the most frequent fungal orders identified were Hypocreales (highest frequency at location M: 22.92%), Agaricales (highest frequency at site F: 19.39%), Helotiales (highest frequency at location F: 19.72%), and Pleosporales (highest frequency at sampling location SH: 13.91%; Figure 2C). The most abundant fungal families detected were *Nectriaceae* (highest frequency at site M: 20.94%), *Inocybaceae* (highest frequency at location F: 17.92%), *Chaetomiaceae* (highest frequency at site G: 5.14%), and *Helotiales* (highest frequency at site B: 4.33%; Figure 2D). *Inocybe* (22.99%), *Fusarium* (48.37%), *Trichocladium* (7.80%), *Phialocephala* (3.27%), *Chalara* (2.69%), *Oidiodendron* (3.20%), *Annulohypoxylon* (0.81%), *Amanita* (1.07%), *Geopora* (2.05%), and *Hymenoscyphus* (2.86%) were detected as the most abundant fungal genera across sampling sites (Figure 2E). The 10 most frequent species were *Trichocladium asperum* (7.80%), *Chalara clidemiae* (1.04%), *Amanita simulans* (0.85%), *Hymenoscyphus menthae* (2.65%), *Fusarium solani* (14.11%), *Cenococcum geophilum* (4.10%), *Pezizomycotina* sp. (4.77%), *Alternaria alternate* (5.64%), *Preussia africana* (3.11%) and *Setophoma terrestris* (3.04%; Figure 2F). The Venn diagram showed unique and core fungal OTUs among all sampling locations. The core fungal taxa (487 OTUs) represented 0.35% of the total fungal OTUs. The highest and lowest number of unique OTUs were detected in the K and B sampling sites, respectively (Supplementary Figure S4).

### Alpha diversity

Based on the results of the four alpha diversity parameters, ACE, Chao1, observed species, and PD, the highest community diversity was observed at the Y sampling location, whereas the lowest alpha diversity indices were present at sampling site G, where these four indices were also found to be statistically different between these two sampling locations as indicated by the Tukey–Kramer HSD test (*p* < 0.0001; Table 1). Wilcoxon rank-sum tests were also applied to evaluate significant differences between groups of sampling locations for each alpha diversity parameter (Supplementary Table S3).

**Table 1:** Alpha diversity indices of rarefied data.

SL^*^	ACE	Chao1	GC^**^	OS^***^	PD^****^	Shannon	Simpson
B	1226.7 (149.65)^bc^	1234.38 (136.38)^b^	0.994 (0.001)^a^	1016.25 (123.82)^bc^	167.4 (22.4)^cd^	7.00 (0.41)^abc^	0.97 (0.01)^a^
F	1308.34 (354.70)^bc^	1,286 (306.84)^b^	0.993 (0.002)^a^	1057.4 (247.42)^b^	173.52 (42.82)^cd^	6.24 (1.12)^d^	0.91 (0.08)^b^
G	1077.13 (85.35)^c^	1081.44 (79.77)^b^	0.994 (0.0007)^a^	849.3 (81.7)^c^	144.28 (14.18)^d^	6.59 (0.60)^bcd^	0.96 (0.02)^a^
K	1757.65 (363.12)^a^	1709.7 (363.03)^a^	0.99 (0.0007)^b^	1376.92 (282.58)^a^	237.67 (33.38)^a^	7.20 (0.70)^a^	0.97 (0.01)^a^
M	1320.62 (105.4)^bc^	1305.32 (96.03)^b^	0.994 (0.001)^a^	1124.43 (101.87)^b^	217.72 (22.44)^ab^	6.97 (0.45)^abc^	0.96 (0.02)^a^
SH	1226.7 (192.63)^bc^	1248.8 (205.57)^b^	0.993 (0.001)^a^	1021.3 (151.17)^b^	193.85 (22.4)^bc^	6.50 (0.86)^cd^	0.95 (0.009)^ab^
Y	1835.9 (146.16)^a^	1797.68 (138.6)^a^	0.99 (0.001)^b^	1414.36 (129.8)^a^	243.57 (23.57)^a^	7.15 (0.52)^ab^	0.97 (0.01)^a^

* Sampling Locations; ** Good’s Coverage; *** Observed species; PD**** Phylogenetic diversity.

For each alpha diversity index, values with different letters differed Significantly (*p* < 0.05) using Tukey’s post hoc test. Values are expressed as mean (standard deviation). K, Meishan to Ping Ke Xian; SH, Taihang Mountains; M, Meishan region; Y, Ya’an area; B, boundaries between forests and grasslands at SNFP; F, forests at SNFP; G, grasslands at SNFP.

### Beta diversity

**Figure 3:**
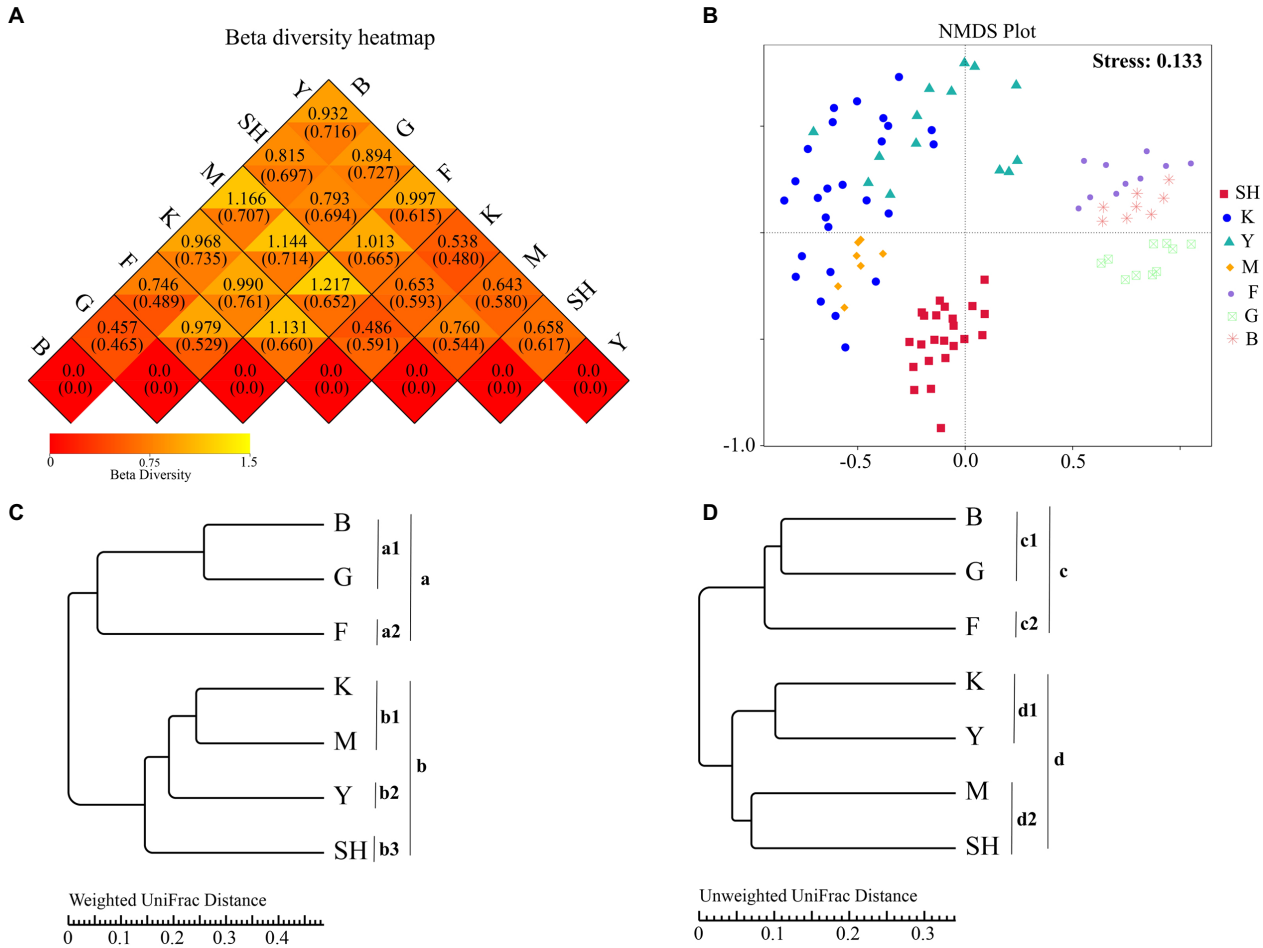
Beta diversity analyses **(A)**, heatmap of beta diversity index, **(B)** two-dimensional NMDS ordination, and **(C)** and **(D)** hierarchical clustering weighted UniFrac distance and Unweighted UniFrac distance of soil fungal communities inferred from ITS-2 rRNA gene at the phylum level for all seven sampling locations based on the Bray-Curtis distance, respectively. A closer distance on the NMDS graph indicates a more similar OTU composition. Abbreviations of variables are given in Figure 1.

To determine the trends in similarities and differences in the community structure at different sampling sites, beta diversity was analyzed *via* weighted–unweighted UniFrac cluster analyses. Weighted UniFrac results (Figure 3A; upper triangles) showed the highest beta diversity value (1.217) between forests in the SNFP and M sampling sites in Sichuan Province, whereas the lowest beta diversity value (0.457) was found between the boundaries of forests (the B sampling location) and grassland. Unweighted UniFrac results (Figure 3A, lower triangles) showed that the highest beta diversity value (0.761) was between grassland and K sampling sites, whereas the lowest beta diversity value (0.465) was found between grassland and the boundary between forest and grassland habitats. NMDS ordination of fungal communities sampled from 98 collection sites within different habitats was performed based on the Bray–Curtis dissimilarity (Figure 3B). The goodness of ordination measure of the NMDS plot was calculated as 0.133, which was below 0.20, indicating the best fit for the data. The results verified clear segregation between fungal taxa compositions in each geographical region. Additionally, a comparison of sampling sites showed that soil fungal communities were statistically different, as indicated by pairwise comparisons of sampling within locations using different statistical procedures, including ADONIS (Supplementary Table S4), and multi-response permutation procedure (MRPP: Supplementary Table S5) analyses. This statistical difference was also depicted by clustering and NMDS analyses. NMDS results also confirmed that the OTU compositions at the SNFP location were clearly separable into three groups (Figure 3B). According to all the above-stated statistical tests at SNFP, the boundary between forest and grassland (B sampling site) statistically differed from the forest region (Supplementary Tables S4, S5). The UPGMA cluster tree structure, based on weighted and unweighted UniFrac distance at the phylum level, showed that fungal OTU compositions were grouped into two main clusters (Figures 3A,D). The weighted UniFrac algorithm showed that the three main sampling sites at SNFP formed a separate group (cluster “a”), whereas the sampling sites in south China and the Taihang Mountains (SH) formed another group (cluster “b”). Further analysis revealed that clusters “a” and “b” were divided into two and three subclusters, respectively (Figure 3C). The unweighted UniFrac clustering results were in line with weighted UniFrac analysis results at the SNFP and formed cluster “c”, composed of two subclusters (Figure 3D). However, the unweighted UniFrac results of sampling sites from south China and the Taihang Mountains showed a different pattern than those from weighted UniFrac results, suggesting that K and Y collection sites formed a separate group from that of M and SH sampling sites (Figure 3D). LEfSe analysis, an algorithm for high-dimensional biomarker discovery, was performed with pooled data, using the rank-sum test, LDA (linear discriminant analysis), to detect OTUs, the abundance of which varied between sampling locations and thus could be used as biomarkers. In total, 59 biomarkers with an LDA score > 4 were identified in taxa ranging from phylum to species. These discriminative fungal taxa could be identified using two features: LDA value distribution histogram (Supplementary Figure S5A) and phylogenetic distribution (Supplementary Figure S5B). The forest region at the SNFP collection site with 16 fungal taxa and the K sampling site with one fungal taxon were significantly enriched in the highest and lowest fungi groups, respectively. LEfSe analysis highlighted that the number of discriminative taxa at the SH, G, Y, M, and B sampling sites were 2, 6, 8, 12, and 14, respectively. Interestingly, enriched taxa in the forest and the boundary between the forest and grassland in SNFP showed a diverse array of fungal taxa ranging from phylum to species level (Supplementary Figure S5A).

### Influence of geographical coordinates on fungal diversity

**Figure 4:**
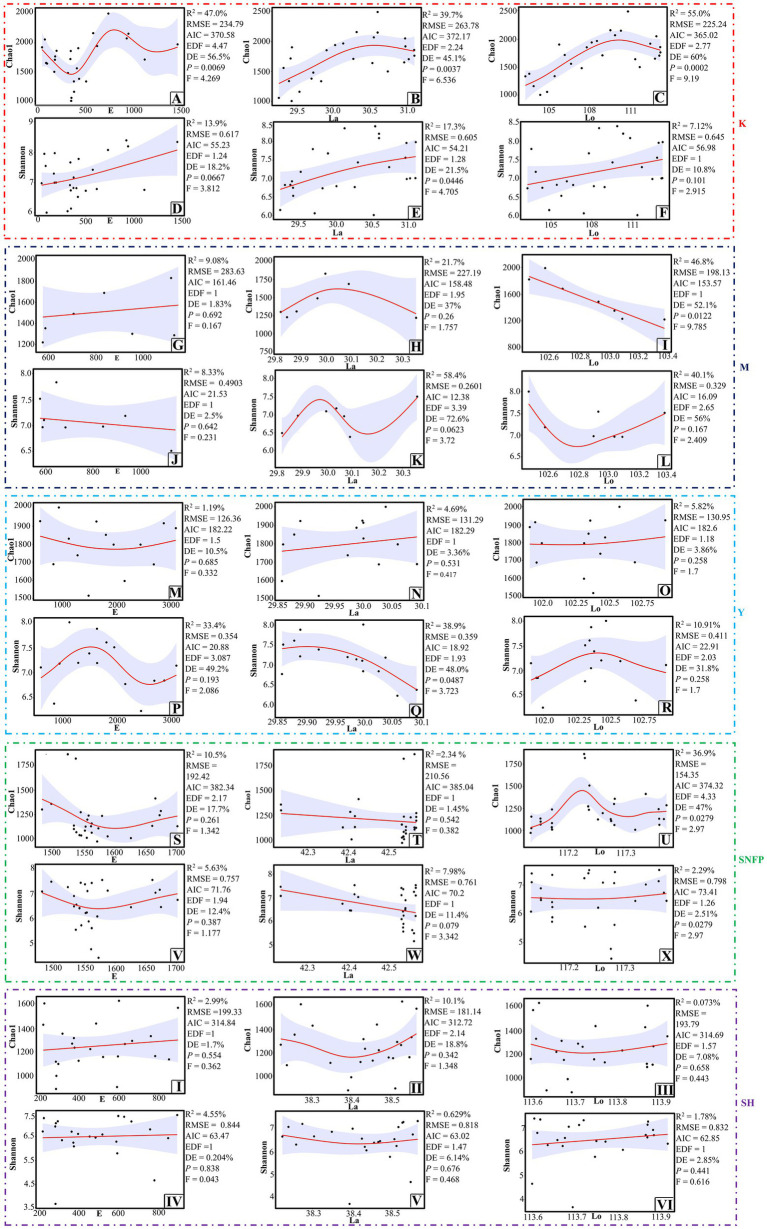
Test of the GAM analysis for detection of an association between fungal richness and diversity with geographical coordinates at the K sampling site **(A–F)**, at the M site **(G–L)**, at the Y sampling site **(M–R)**, at the SNFP site **(S–X)**, and the SH sampling site **(I–VI)**. 95% confidence interval is highlighted with the light violet color. Abbreviations of variables are given in Figure 1.

Fungal richness was significantly associated with the geographical coordinates (Figures 4A–C), but fungal Shannon diversity was statistically linked with just longitude at the K sampling site. In this region, increased fungal diversity was observed with increasing longitude (Figure 4E). Fungal richness (Figure 4I) and Shannon diversity were significantly associated with longitude and latitude at the M and Y sampling locations, respectively (Figure 4Q). In both locations, decreased fungal richness and diversity were observed with increasing either longitude or latitude. At the SNFP location, the fungal richness and other alpha diversity indices were considerably associated with longitude and different patterns. Fungal richness peaked at the highest level when the longitude was a bit more than 117.2°E, whereas fungal diversity followed a relatively stable trend with increasing longitude. There was no evident association between fungal Shannon diversity and richness with geographical coordinates at the Taihang Mountains, suggesting that besides the influence of geographical properties, other environmental variables can shape fungal communities. Fungal community dissimilarity was significantly associated with geographical coordinates at the Y sampling sites. Besides, a significant link was observed between longitude with fungal community dissimilarity at the SH sampling site (Supplementary Figure S6).

**Figure 5:**
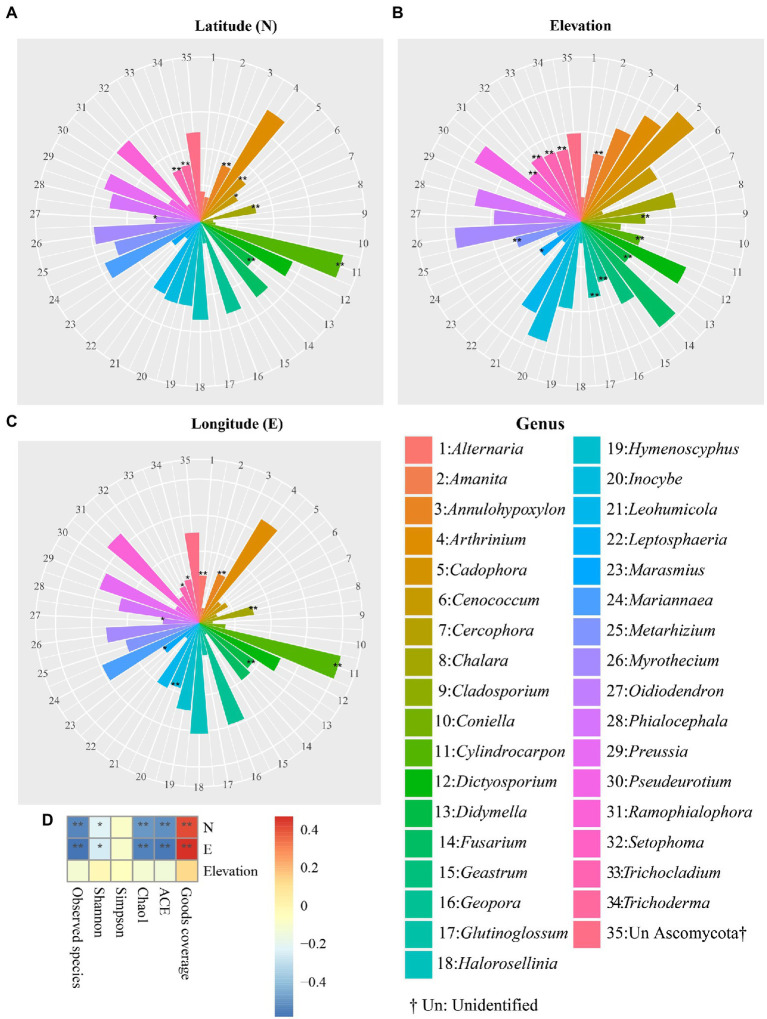
The correlation between geographical coordinates and predominant genera. Congruence of abundant fungal genera with latitude **(A)**, elevation **(B)**, longitude **(C)**, and alpha diversity indices **(D)**. The correlation heatmap was used to show the relationship between abundant fungal genera and GPS information by evaluating the correlation coefficient between each fungal genus with each GPS coordinate. Asterisks denote statistically significant Spearman analysis * and ** represent *p*-values ≤ 0.05 and 0.01, respectively. The deepest blue (−0.4) and red (0.4) represent the negative and positive congruence of alpha diversity parameters with GPS coordinates, respectively.

The effect of geographical coordinates, including latitude (N), longitude (E), and elevation on the relative abundances of dominant genera, was also measured using Spearman rank correlation coefficients (Figure 5). The Spearman results showed that the relative abundances of nine fungal genera, *Oidiodendron* (*p =* 0.0143), *Trichocladium* (*p =* 0.0022), *Trichoderma* (*p =* 0.0015), *Annulohypoxylon* (*p =* 0.0008), *Cadophora* (*p =* 0.0012), *Cenococcum* (*p =* 0.0201), *Chalara* (*p =* 0.0019), and *Didymella* (*p =* 0.0003) were statistically and positively congruent with latitude. In addition, longitude was significantly and positively associated with the relative frequency of dominant fungal genera, including *Alternaria* (*p =* 0.0085), *Annulohypoxylon* (*p =* 0.0032), *Chalara* (*p =* 0.0019), *Didymella* (*p =* 0.0001), *Inocybe* (*p =* 0.0001), *Marasmius* (*p =* 0.0155), and *Oidiodendron* (*p =* 0.0443). The frequencies of 12 abundant fungal genera, such as *Amanita* (*p =* 0.0021), *Cladosporium* (*p =* 0.0042), *Cylindrocarpon* (*p =* 0.0062), *Didymella* (*p =* 0.0079), *Geopora* (*p =* 0.0052), *Glutinoglossum* (*p =* 0.0006), *Marasmius* (*p =* 0.0272), *Metarhizium* (*p =* 0.0028), *Ramophialophora* (*p =* 0.0029), *Setophoma* (*p =* 0.0005), *Trichocladium* (*p =* 0.0009) and *Trichoderma* (*p =* 0.001), were positively and significantly correlated with elevation. In addition, Spearman correlation analysis demonstrated that none of the alpha diversity indices was significantly correlated with elevation. Furthermore, the Good’s coverage index of alpha diversity showed positive and significant congruence with latitude and longitude. ACE, Chao1, Shannon, as well as observed species indices were negatively correlated with longitude and latitude (Figure 5D). No relationship was found between the Simpson index and either latitude or longitude. In addition, the Spearman correlation was evaluated from phylum to species level (Supplementary Figures S7-S11). Spearman correlation analysis at the phylum level indicated that the relative frequency of Blastocladiomycota was significantly and positively correlated with elevation (*p =* 0.0412; correlation = 0.2065) as well as longitude (*p =* 0.0458; correlation = 0.2021), whereas the relative frequency of the Rozellomycota was significantly but negatively correlated with longitude (*p =* 2.5236E-09; correlation = −0.5572) and latitude (*p =* 1.6795E-09; correlation = −0.5623; Supplementary Figure S7). At the species level, Spearman correlation indicated that *Cadophora finlandica*, *Preussia Africana*, unknown species from Pezizomycotina, and *Trichocladium asperum* were correlated with elevation, latitude, and longitude at different intensities (Supplementary Figure S11).

### Fungal co-occurrence patterns

**Figure 6:**
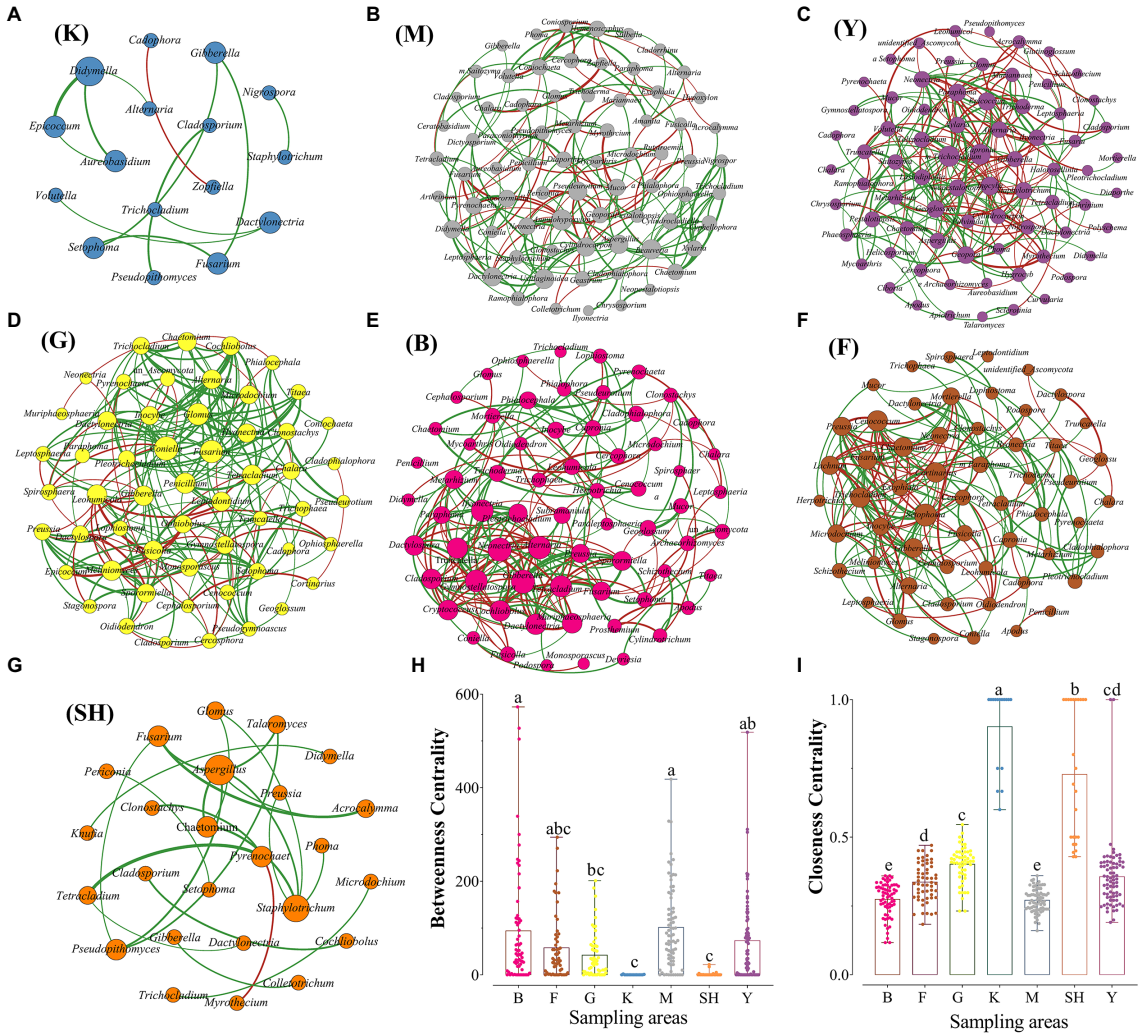
The site-specific co-occurrence network maps of the fungal community at the genus level in (**A** = K) Meishan to Ping Ke Xian; (**B** = M) Meishan region; (**C** = Y) Ya’an area; (**D** = G) grasslands at SNFP; (**E** = B) boundaries of forests and grasslands at SNFP; (**F** = F) forests at SNPF; and (**G** = SH) the Taihang Mountains. The values of betweenness centrality **(H)** and closeness centrality **(I)** were calculated as two main features of co-occurrence networks. Red links indicate negative interactions between the two individual nodes, whereas green links indicate positive interactions.

The fungal co-occurrence network captured 12 (K sampling location with 16 nodes), 23 (SH sampling location with 25 nodes), 181 (M sampling location with 74 nodes), 237 (Y sampling location with 75 nodes), 228 (B sampling location with 66 nodes), 183 (F sampling location with 56 nodes), and 215 (G sampling location with 55 nodes) edges, including positive and negative relationships (Table 2; Figure 6). Our network features showed that specific fungal genera were adapted in each sampling location based on the high value for a topological feature like “degree.” In the K sampling site, nodes with high degree values were mostly linked with the *Didymella* genus. In the Y sampling location, two genera of *Trichocladium* and *Capronia* received the high degree values, whereas, in the M sampling location, the high degree value was primarily affiliated with the member of the *Annulohypoxylon* genus. Nodes with high degree values were mainly linked with *Gibberella* and *Neonectria* at the B sampling location. In contrast, nodes with high degree values were primarily affiliated with *Coniella* and *Setophoma* genera members at the G and F sampling locations, respectively. The *Aspergillus* genus received a high degree value at the SH sampling location. To illustrate the distance between two members and the complexity of the network, two network features, such as the average path length and average degree, were measured. The highest average degree was recorded for the B sampling location, indicating that the soil fungal community relationship was more complex than that of other sampling locations. The highest average degree was recorded for the G sampling location, meaning that the soil fungal community was more intense in the grassland habitat. Interestingly, the highest number of positive (co-presence) correlations were inferred from the G sampling location, indicating that the presence of enough natural resources like nutrients lead soil fungal community to commensalism relationships. In contrast, the highest number of negative (mutual exclusion) relationships were inferred from the Y sampling location, indicating a high possibility for increased ecologic niche differentiation because of a high proportion of competition among soil fungal communities due to the scarcity of natural resources like nutrients (Ji et al., 2019). Based on the average clustering coefficient, soil fungal community associations were more tightened at the K sampling location, whereas soil fungal community was less concentric at the M sampling location (Table 2). Key nodes of the different sampling locations were identified based on the calculation of the betweenness centrality (BC) feature. Interestingly, the highest value for the BC feature was recorded among all sampling sites from the B sampling location, included *Herpotrichia*, *Muriphaeosphaeria*, *Capronia*, *Apodus*, *Cercophora*, *Trichoderma*, *Titaea*, *Inocybe*, and *Trichophaea*, specifying these genera were the key connectors for the fungal network in the B sampling location, whereas the lowest BC values were observed from the K and SH sampling locations, indicating that these fungal genera had a more peripheral location for the fungal network in the specified sites (Supplementary Table S6).

**Table 2:** Topological features of site-specific co-occurrence network in Figure 6.

	K	SH	M	Y	B	F	G
Nodes	16	25	74	75	66	56	55
Edges	12	23	181	237	228	183	215
Average degree	1.5	1.84	4.892	6.32	6.909	6.536	7.818
Average weighted degree	1.028	1.24	4.108	4.34	5.541	4.788	5.72
Network diameter	2	3	10	8	12	8	6
Graph density	0.1	0.077	0.067	0.085	0.106	0.119	0.145
Modularity	0.774	0.678	0.587	0.411	0.463	0.426	0.418
Connected components	6	7	1	2	1	1	1
Average clustering coefficient	0.762	0.547	0.39	0.405	0.488	0.498	0.539
Average path length	1.2	1.85	3.784	3.092	3.904	3.11	2.57
Positive interactions	15	24	124	126	133	115	168
Negative interactions	1	1	57	111	95	68	47
Radius	1	1	5	1	6	4	4
Sum changes	0.0039	0.0021	0.0114	0.0023	0.004	0.0029	0.0056
Total triangles	2	5	138	288	408	272	313

K, Meishan to Ping Ke Xian; SH, Taihang Mountains; M, Meishan region; Y, Ya’an area; B, boundaries between forests and grasslands at SNFP; F, forests at SNPF; G, grasslands at SNFP.

### Functional fungal community diversity (FUNGuild)

**Figure 7:**
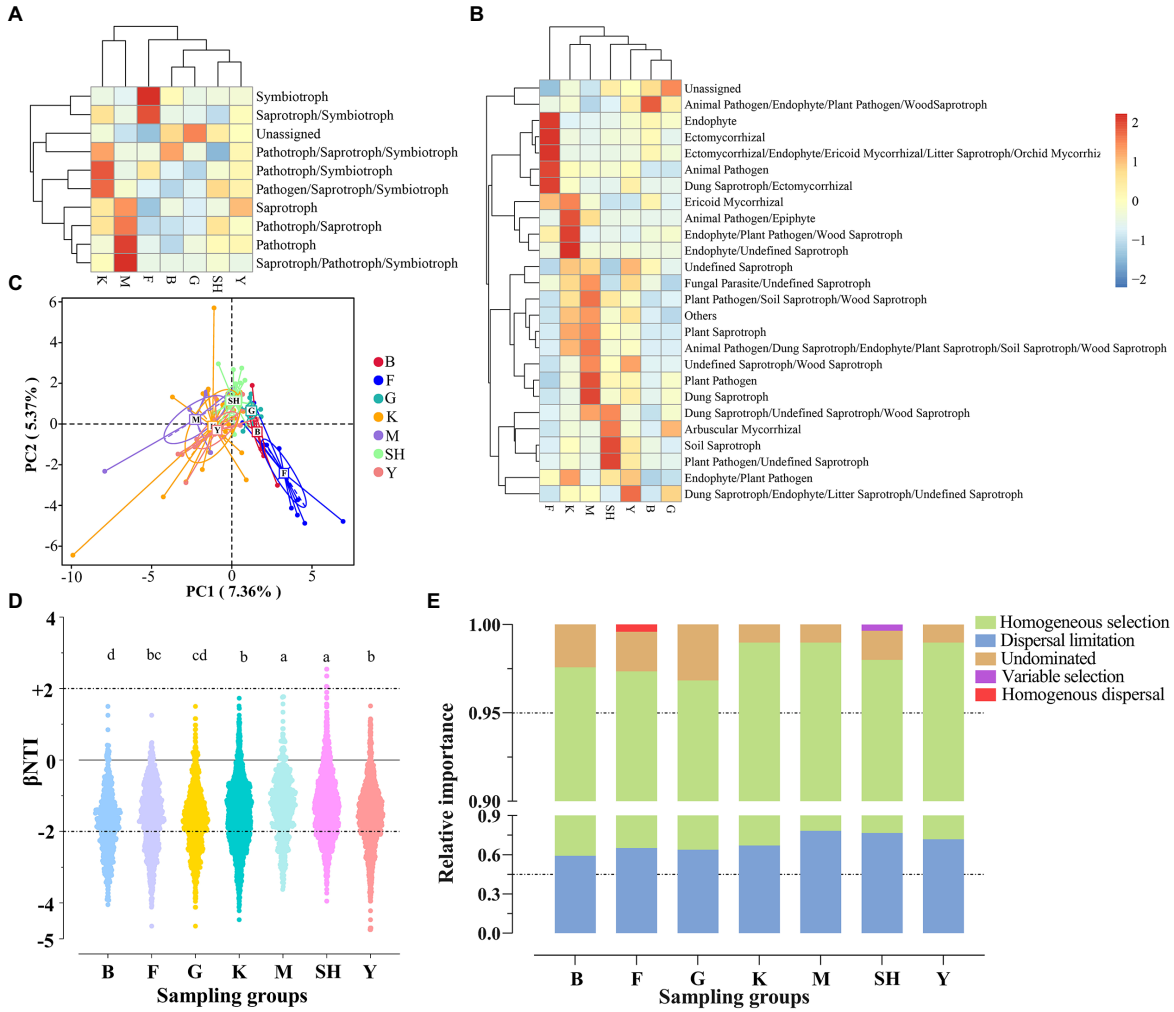
Assignment of the functional guild (functional group) using FUNGuild software. The FUNGuild ver 1.0 database was used to assign ecological functions, trophic modes **(A)**, and guilds **(B)** to each OTU. Variations of guilds among sampling sites **(C)** based on principal component analysis (PCA). Null model analysis reveals the value of βNTI **(D)** and the assembly mechanism of fungal communities across different sampling locations **(E)**. Abbreviations of variables are given in Figure 1. Lowercase letters significantly differ above each sampling group at *p* < 0.05.

Among functional modes, symbiotroph and saprotroph-symbiotroph were significantly more abundant in their relative abundance in the forest habitats at the SNFP site. In addition, the unassigned mode was statistically (*p* < 0.05) higher in the G sampling site (Figure 7A). Similarly, when the fungal taxa belonging to various ecological guilds were compared, the functionally “unassigned” fungal group showed a significantly (*p* < 0.05) higher relative abundance at the G collection site. In addition, the results of FUNGuild showed that soils from the Saihanba forest were overwhelmingly dominated by taxa belonging to the symbiotroph group, and guilds such as endophyte, ectomycorrhizal, ericoid, and orchid mycorrhizal groups were abundant at the F collection site (Figure 7B). Additionally, Pathotroph and saprothroph-pathotroph-symbiothroph were the most abundant life strategies at the M collection site. We further used principal component analysis (PCA) to evaluate the dissimilarities of fungal guilds among different sampling sites. The results showed that the guilds of soils from the Saihanba forest had a certain degree of differentiation compared with other sampling sites (Figure 7C).

### Fungal community assembly processes

The βNTI (β-nearest taxon index) distributions differed significantly across different habitats (*F*_6, 9,506_ = 55.2148, *p* < 0.0001; Figure 7D). Null model analysis showed that the relative contribution of deterministic (|βNTI| ≥ 2) and stochastic (|βNTI| < 2) processes in various habitats differed greatly. The stochastic process of dispersal limitation and homogenous dispersal were the major assembly processes for fungal communities in different habitats (average: 60.29%), whereas the deterministic processes of variable selection and homogenous selection were the minor assembly processes for fungal communities in different habitats (average: 25.63%; Figure 7E).

## Discussion

The current study underlines the soil fungal community’s natural distribution and community assembly processes from different ecosystem types and soil fungal community co-occurrence network using 490 soil samples taken from 98 forest and grassland sites spanning the Sichuan, Hubei, and Hebei Provinces of China, *via* high-throughput analysis of the ITS-2 region for fungal delimitation. Based on our results, we conclude that the fungal assembly pattern was mostly driven by dispersal limitation than selection in different habitats. However, fungal alpha and beta diversities exhibited different biogeographical patterns. Besides, we show that ecosystem-related effects on soil fungal communities can be consistently observed across a regional scale.

### Fungal community patterns change across sampling regions

Our findings suggested that ecosystem types and geographical distances effectively shape soil fungal communities by significantly changing the overall richness of taxa (Figure 2). This outcome aligns with other studies in which different ecosystem types could impact soil fungal taxa (Thomson et al., 2015; Szoboszlay et al., 2017; George et al., 2019). The most abundant fungal phylum was found to be Ascomycota, suggesting its high ecological importance in soils (Wang et al., 2021). In contrast, the three most prevalent classes were Sordariomycetes, Dothideomycetes, and Leotiomycetes (Figure 2). Sordariomycetes consist of several prominent human and plant pathogens (De Hoog et al., 2000; Murgia et al., 2019). High frequencies of the genus *Fusarium* (class Sordarimycetes) were detected in the southern regions and the Taihang Mountains of north China, indicating that this vast and complex genus was widely distributed. This genus, which induces a diverse array of plant diseases (He et al., 2021) and produces several mycotoxins, is also a known human pathogen (Summerell et al., 2010). Our results showed that the distribution and occurrence of fungal genera such as *Fusarium* and *Chalara* in warmer climates (Sichuan forests) were different and higher than those in cold temperatures such as SNFP. The largest and most ecologically diverse class of Ascomycota was Dothideomycetes (a potential oligotrophic class), containing over 19,000 species of saprotrophs, parasites, and occasional lichen-forming types, which essentially function in nature and the carbon cycle as degraders of plant biomass (Hyde et al., 2013; Taylor et al., 2015). Dothideomycetes were the second most frequent class in all sampling sites (Figure 2). The distribution and occurrence of Dothideomycetes are recorded in different ecosystems ranging from hot deserts (Murgia et al., 2019) to Antarctica (Ruibal et al., 2009; Selbmann et al., 2013). Thus, the wide range of Dothideomycetes distribution indicated that oligotrophic status is one of the common fungal functional groups across our sampling areas. We found that some soil fungal diversification and distribution were dominated by a defined geographical location (OTU-area relationship), indicating that they were not diverse in occurrence and distribution. Perhaps, the environmental gradients could drive fungal abundances by altering, in particular, fungal taxa with a specific function (Pellissier et al., 2014). For example, our results showed that Ectomycorrhiza *Inocybe* (Agaricomycetes) was the most abundant genus at site F in the cold temperate region, but it was a key node for the Y and B sampling locations (Supplementary Table S6). This result reflected that of Shi et al. (2014), who found a high abundance of this genus in certain Chinese forest biomes. In addition, it has been highlighted that the frequency of Agaricomycetes, which is due to its exertion of several significant ecological effects such as ectomycorrhizal symbiosis and wood-decay in shrubs, perennial alpine plants, and trees, was emphatically linked with the richness of plants (Blaschke, 1991; Hibbett et al., 2014). The comparison of the high frequency of Agaricomycetes at site F with those of other studies confirms that Agaricomycetes were indispensable for the ecosystem functioning, not only in forest soil (Peay et al., 2007; Clemmensen et al., 2013) but also in grassland soils (Pellissier et al., 2014). Similarly, the genus *Inocybe* was dominant in the border between forest and grassland, whereas the genus *Oidiodendron* displayed a high frequency in the biological community of the grassland. It has been previously reported that the abundance of the genus *Inocybe* was associated with the growth of either conifers or broad-leaved trees (Puschner, 2018). The Venn diagram developed by the current study showed that the number of non-shared OTUs in forest soil from the Sichuan and Hubei Provinces was higher than that from the SNFP site (Supplementary Figure S4). Two crucial facts may explain this result. First, the forest canopy in Sichuan and Hubei Provinces mainly consists of mixed plant species with high heterogeneity, whereas the forest vegetation in SNFP is mostly composed of a monoculture canopy. More recent evidence suggests that the soil fungal community in mixed forests shows a higher level of diversity than in pristine forests, indicating that microbial species in mixed plantation environments place higher in richness and diversity (Jiang et al., 2012; Wu et al., 2019). Secondly, the mean annual temperature at SNFP was −1.4°C with a long cold winter, whereas the mean temperature in Sichuan and Hubei Provinces was ~8–10°C (http://data.cma.cn/data/online/t/1: in Chinese). Therefore, the higher number of unique OTUs seen in southern forest regions may be attributed to a combination of forest vegetation types and climate (temperature). The lowest, and highest number of non-shared OTUs in the SNFP was recorded in grassland (134 OTUs) and forest (186 OTUs) habitats, respectively (Supplementary Figure S4). Naturally, forest regions grow in multiple layers in comparison with grassland habitats with less dense vegetation, indicating the high spatial heterogeneity of multi-layered vegetation of forests may potentially lead to the high degree of diversity and richness seen in fungal community compositions (Baldrian, 2016; Erdős et al., 2019).

The UPGMA trees at the phylum level indicated that fungal OTU compositions from boundary regions were clustered with those from grassland habitats (cluster “a_1_”), whereas forest samples formed a separate group (cluster “a_2_”; Figure 3C). This result indicated that fungal OTU compositions of boundary areas might be similar to those of forest exteriors. Furthermore, weighted UniFrac ADONIS and MRPP analyses showed that the fungal community composition in boundary areas was not significantly different from that in grassland habitats (Supplementary Tables S4, S5). A possible explanation for fungal taxa from site B clustering with those from site G may be that the forest regions in SNFP were not distinctly segregated from grassland habitats. Therefore, it is impossible to treat fungal compositions at the edges of our sampling sites as separate communities. We should mention that the clustering sites B, G, and F together can be explained due to their close proximity to each other, and it is expected that more similar local climate and/or soil conditions coupled with the relatively high dispersal potential of fungal taxa.

### Impact of geographical coordinates on fungal diversity variation

Our Spearman analyses indicated that none of the alpha diversity indices were statistically correlated with elevational effects (Figure 5D), somehow suggesting a lack of elevational pattern in soil fungal community at an altitudinal gradient from 43 to 3,100 m in studied areas. This apparent lack of correlation can be attributed to reasons such as different methods for analyzing the diversity of microbial communities that may impact the measurements of fungal distribution patterns along elevations (Ni et al., 2018; Chen et al., 2022). Also, the elevational gradient comprises different plant types, which is expected to influence soil fungal community diversity patterns along elevations (He et al., 2017; Yang et al., 2017). Shen et al. (2014) showed a lack of elevational patterns in soil fungal diversity at the elevational gradient from 530 to 2,200 m on Changbai Mountain. Almost 4 years later, Ni et al. (2018) analyzed the data from the tundra soils of Changbai Mountain and concluded that elevation strongly influenced fungal communities’ diversity, where diversity increased linearly with increasing elevation. Unlike Spearman analysis, the associations of geographical coordinates with fungal alpha diversity indices (Figure 4) and fungal community dissimilarity (Supplementary Figure S6) were regionally assessed using the GAM analysis. The results showed that the impact of geographical coordinates on each alpha diversity index and fungal community dissimilarity differed from region to region. Several reports showed that fungal diversity did not follow a general spatial distribution pattern in line with our results. For instance, Tedersoo et al. (2014) and Liu J. et al. (2015) highlighted that fungal diversity decreased with increasing latitude, whereas Shi et al. (2014) found that fungal diversity peaked at mid-latitudes and descended toward high and low latitudes. The combination of several substantial *factors plays a role* in the lack of a consistent spatial distribution pattern for fungal diversity, such as organizational levels of interest (Liu L. et al., 2015), spatial scales (Bahram et al., 2015), ecosystem or land-use types (Monard et al., 2013), and the environmental settings (Hawkes et al., 2011). To date, several studies have investigated the latitudinal distribution of soil fungi across Chinese habitats. For instance, Shi et al. (2014) highlighted a unimodal distribution pattern of soil fungal communities in western China’s forests, which peaked at the middle latitude (temperate forests), which was led mainly by temperature. Liu J. et al. (2015) reported the occurrence of fungal community diversity along a latitudinal gradient in the black soil zone of northeastern China. In the current study, except for Good’s coverage index, all statistical analyses indicated that the four alpha diversity indices were negatively linked with latitude. Furthermore, as stated earlier, site Y, which had the lowest altitude (29°N) compared to other sampling locations, displayed the highest OTU richness. These results are substantiated by other studies, showing negative congruence between latitude and the species richness of different types of organisms (Helmut, 2004), including endophytic fungi (Arnold, 2007), mammals (Ceballos and Ehrlich, 2006), amphibians (Buckley and Jetz, 2007), marine bacteria (Pommier et al., 2007), and vascular plants (Kier et al., 2005). However, our results contradicted those of a recent study in which latitude was unimodally associated with species richness of fungi at the boundary between subtropical and temperate zones in China, with richness peaking around mid-latitude (35°N; Wang et al., 2019). According to our results, spatial variation was apparent in the composition of fungal OTUs among sampling sites (Figure 3B), indicating that the similarities between fungal communities decreased greatly with geographical distance and emphasizing the importance of dispersal in fungal community structures (Li P. et al., 2020). Several studies have highlighted the influence of forest vegetation on the composition of fungal communities, suggesting two main features: first, the fungal composition of a specific forest biome cannot be generalized as corresponding to multiple arrays of forest ecosystems; secondly, there is robust congruence between fungal and plant diversities (Baldrian, 2016; Liu et al., 2021; Qu et al., 2021).

### Network-level topological features vary across geographical regions

The co-occurrence network-based analysis was carried out using fungal genera to determine the habitat patterns of topological features across three regions of China. The results showed that topological features were different between the southern and northern areas. Our results indicated that soil fungal communities’ diversity was higher at the high spatial heterogeneity of multi-layered vegetation of southern forests in comparison with the grassland habitat. However, the network visualization revealed that fungal interactions were more intense (stronger relationships) in the grassland habitat with the highest number of positive correlations, which suggests the grassland fungal communities had strong niche specialization and thus did not support our first hypothesis (H_1_). Its network was predominantly co-operative, with probably less competition. However, we should note that the interferences of fungal genera’s interactions from the correlation relationship can be imperfect because the abundance of genera does not inevitably indicate causal relationships (Li L. et al., 2020). As further shown in Supplementary Table S6, genera typifying the boundary of forest and grassland at SNFP from the northern region had higher BC values in comparison with genera typifying the southern regions, indicating the importance of key nodes that specific fungal genera use over the interactions of other fungal genera in the network. In contrast, the low BC value represented those fungal genera, which were located away from the network’s core, suggesting the low impact of such taxa on other fungal interactions in the community (Ma et al., 2016). We were aware of the fact that network analysis is not a full remedy. Therefore, further experimental studies are needed to disentangle the leading biotic and abiotic drivers shaping soil fungal communities in different habitats.

### Functional predictive analysis of fungal communities

ITS-based FUNGuild bioinformatic tool (using a community-annotated database based on the taxonomic assignment) provides a sufficient explanation to identify fungal functional groups across our sampling sites. More specifically, the FUNGuild analysis assigned the fungal OTUs to the main trophic modes: pathotrophs, symbiotrophs, and saprotrophs (Figure 7A). The results suggested that the effects of different habitat types on soil fungal functional profiles can be geographical location-dependent. Even our results indicated that the relative abundances of saprotrophs (*F*_6, 97_ = 1.5353, *p* = 0.1756) and pathotrophs (*F*_6, 97_ = 1.3161, *p* = 0.2581) did not significantly differ among sampling sites, but the relative abundance of saprotrophic lineages was higher in Sichuan forests. This result suggests the vital role of these critical decomposers of soil organic matter in multilayer forest regions (Yuste et al., 2011). Besides, our results showed that sampling locations impacted the relative abundance of symbiotrophs significantly (*F*_6, 97_ = 9.9532, *p* < 0.0001). From this functional group, the proportion of ectomycorrhizal symbiotroph lineages was much higher in the forest region of SNFP than in other sampling sites. This result may be explained by the fact that ectomycorrhizal fungi seemed to have been involved as an adaptation mechanism in the boreal forest (Qu et al., 2021). The difference in the relative abundance of all guild-assigned fungi in our sampling sites from various geographic gradients can be explained by several reasons. The existing evidence has highlighted that the changes in precipitation, temperature, climate, and other factors induced by geographical location might have a negligible influence on the abundance levels of particular fungal taxa, whereas the host selectivity as a biotic component of the local environment can play more principal functions in forming fungal trophic groups (Davison et al., 2016).

### Geographical patterns of fungal community assembly processes

By using an ecological null modeling approach, this study investigated the ecological assembly processes influencing fungal community structure in different habitats. Stochastic and deterministic processes structure microbial communities differently, and their relative role in shaping soil fungal communities is currently being debated (Zhou and Ning, 2017). The levels observed in our investigation are different from those observed by Fierer and Jackson (2006), Lozupone and Knight (2007), Dumbrell et al. (2010), Wang et al. (2016), Zhou et al. (2016), Zhang et al. (2019), Zheng et al. (2020, 2021), who reported that the assembly of microbial communities relies predominantly on deterministic processes (niche-based) operated by contemporaneous environmental variables such as nutrients, salinity, precipitation, temperature, and pH. Stochastic processes can also be a driver for bacterial communities, which are stimulated by dispersal limitation and geographical segregation (Wang et al., 2013). A clear neutral pattern was observed in our study, which indicates that stochastic processes, particularly dispersal limitation, tended to be more critical in structuring the assembly of the soil fungal community and thus did not support our second hypothesis (H_2_). Bacterial community assembly processes are considered in most recent studies compared with soil fungal community assembly, and the importance of stochastic processes in provoking and maintaining fungal biodiversity is infrequently admired. However, some investigations have highlighted the implication of neutrality for certain fungal communities, arbuscular mycorrhizal (AM) and ectomycorrhizal fungi (EM) in soil and roots (Caruso et al., 2012; Davison et al., 2016; Schröter et al., 2019). Our observation in the context of different habitats is in line with the results of recent large-scale studies in natural soil from different habitats across Scotland (Powell et al., 2015), suburban agricultural districts of Shanghai, China (Li et al., 2021), and a wine-growing region of Italy (Larsen et al., 2022). In contrast to our finding, however, Birch et al. (2022), who reported that deterministic processes, particularly homogenizing selection, dominated fungal communities along an 1,800 km transect of North America across *Pseudotsuga* forests. Our results indicate a greater tendency toward stochastic processes, which is inconsistent with the seminal work of Zheng et al. (2021), who highlighted a substantial divergence among whole fungal communities and the AM and EM fungal community subsets within and among 12 forest ecosystems along a large latitudinal gradient. These findings might provide new evidence for the size-dispersal hypothesis that organisms with larger body sizes, like fungi, are more dispersal-limited than those with smaller body sizes, like bacteria (Zhang et al., 2021; Larsen et al., 2022). The more substantial dispersal limitation in fungal community assembly also supplied a conceivable illustration for the more elevated beta diversities (heterogeneity in species composition; Galiana et al., 2018). This might be a reasonable explanation for the M sampling location with higher beta diversity (weighted UniFrac distance; Supplementary Figure S12) and dispersal limitation than other sampling locations. Our fungal networks showed that the K and SH sampling locations were more modular (Table 2), indicating that fungal taxa occupied a more decentralized niche in these regions (Shi et al., 2016). This discrepancy of fungal communities among different geographical locations and habitats might be likely due to the higher spatial heterogeneity, which is caused by more substantial dispersal limitation (Galiana et al., 2018); subsequently, a low-level exchange of fungal taxa restrains the establishment of local taxa in distant fundamental niches (Stegen et al., 2013). Generally, we expect that more elevated environmental variability can lead to higher microbial structure heterogeneity. However, our results demonstrated that dispersal limitation relatively monopolized the ecological process shaping the fungal community. Previously, it was found that a lack of sufficient nutrients might develop into structural variability in fungal communities, consequently leading to higher dispersal limitations (Li P. et al., 2020). Thus, dispersal limitation as a part of stochastic processes results in patterns of the fungal community composition indiscernible from random assemblages (Chave, 2004). Another parameter that can impact fungal community assembly processes is plant communities through direct host-microbial interactions and rhizosphere effects (Martínez-García et al., 2015) and indirect mediation of soil physicochemical properties (Zak et al., 2003). The results of this study do not explain the impacts of abiotic factors on fungal community assemblies, but some studies showed that fungal community assemblies could be impacted by seasonal and successional variations (Liu et al., 2021; Kang et al., 2022; Wang et al., 2022). Our study’s shortcomings accommodate the paucity of evidence of the soil edaphic parameters and the consideration of other environmental variables like diversity vegetation canopies. Thus, we are unable to clarify the mechanisms behind the predominant role of dispersal limitation across different habitats.

## Conclusion

In this study, our analyses showed a significant effect of different habitats on soil fungal alpha and beta diversities. The result of the null model simulation showed that stochastic processes impacted the outcome of fungal community assembly, a long large-scale geographical gradient in different habitats. Our results showed that fungal Shannon diversity and richness were higher in Sichuan and Hubei Provinces with diverse mixed forests than in mono-culture forests from Hebei Province. Unexpectedly, grassland habitats with low fungal diversity showed more intense fungal correlations than forest ecosystems, suggesting that the higher diversity of fungal taxa in a particular habitat may not be a reliable predictor to infer fungal network complexity. Moreover, our results showed that enriched taxa in the forest and the boundary between the forest and grassland in SNFP contained a diverse array of fungal taxa ranging from phylum to species level, indicating the large-scale, long-term plantation in the manmade forest (SNFP) can help to foster soil fungal community diversity. The current study’s findings may have a number of important implications for future projects aiming to provide a reliable snapshot of soil fungal network complexity and fungal assembly processes in different habitats.

## Data availability statement

All sequence data were previously deposited in the National Center for Bioinformatics Information (NCBI) Sequence Read Archive database under the BioProject ID PRJNA551928, Submission ID SUB5873947.

## Author contributions

AM and HW collected the samples. ChW: data analysis. AM, MW, and CaW designed the project, carried out the experiments, data analysis, and wrote the manuscript. ZY edited the manuscript. AM and JL designed the project, supervised the research, and edited the manuscript. All authors contributed to the article and approved the submitted version.

## Funding

This study benefited from the Hebei Science and Technology Innovation Base Project (216Z2903G), the National Key Research and Development Project of China (2018YFC0506900), and the National Natural Science Foundation of China (32070494). This study was also partially supported by a postdoctoral research grant from Hebei Normal University to AM (211399).

## Conflict of interest

The authors declare that the research was conducted in the absence of any commercial or financial relationships that could be construed as a potential conflict of interest.

## Publisher’s note

All claims expressed in this article are solely those of the authors and do not necessarily represent those of their affiliated organizations, or those of the publisher, the editors and the reviewers. Any product that may be evaluated in this article, or claim that may be made by its manufacturer, is not guaranteed or endorsed by the publisher.

## Footnotes

^1^ http://ccb.jhu.edu/software/FLASH/
