# Supplementary material for: Fungal diversities and community assembly processes show different biogeographical patterns in forest and grassland soil ecosystems
Source: Front Microbiol. 2023 Feb 1;14:1036905. doi: 10.3389/fmicb.2023.1036905 (PMC9928764; doi:10.3389/fmicb.2023.1036905)
Supplement: Supplementary file 2 [file Data_Sheet_1.docx]

**Supplementary Information**

**Fungal diversities and community assembly processes show different biogeographical patterns in forest and grassland soil ecosystems**

Min Wang^†^, Can Wang^†^, Zhijun Yu, Hui Wang, Changhao Wu, Abolfazl Masoudi^*^, Jingze Liu^**^

**Affiliation**:

Hebei Key Laboratory of Animal Physiology, Biochemistry and Molecular Biology, Hebei Collaborative Innovation Center for Eco-Environment, Ministry of Education Key Laboratory of Molecular and Cellular Biology, College of Life Sciences, Hebei Normal University, Shijiazhuang, Hebei 050024, P. R. China

**Address**: College of Life Sciences, Hebei Normal University, 20 Nanerhuan East Road, Shijiazhuang, Hebei Province, P.R. China

**Email addresses:**

Min Wang: [wangmin0811@stu.hebtu.edu.cn](mailto:wangmin0811@stu.hebtu.edu.cn)

Can Wang: [wangcan@hebtu.edu.cn](mailto:wangcan@hebtu.edu.cn)

Zhijun Yu: [yuzhijun@hebtu.edu.cn](mailto:yuzhijun@hebtu.edu.cn)

Hui Wang: [whui1981@163.com](mailto:whui1981@163.com)

Changhao Wu: [wuchangeh@163.com](mailto:wuchangeh@163.com)

Abolfazl Masoudi: [abolfazl.masoudi@hebtu.edu.cn](mailto:abolfazl.masoudi@hebtu.edu.cn)

Liu Jingze: [liujingze@hebtu.edu.cn](mailto:liujingze@hebtu.edu.cn)

To whom correspondence should be addressed: *Corresponding Authors: Tel/fax: +86 311 80787551, [abolfazl.masoudi@hebtu.edu.cn](mailto:abolfazl.masoudi@hebtu.edu.cn) (AM), [liujingze@hebtu.edu.cn](mailto:liujingze@hebtu.edu.cn) (JL)

^†^ These authors contributed equally

**This supplementary information is composed of ‘Supplementary text’, 12 Figures, and 6 Tables.**

**Supplementary Text**

**Supplementary Information:**

**Bioinformatic and statistical analyses**

Adaptors/distal priming sites were removed from Ion-Torrent sequences, keeping a minimum sequence length of 50 base pairs (bp), and filtering the bases whose tail mass value was less than 20. High-quality clean tags were compared with the reference database using the UCHIME algorithm to detect and to remove chimeric sequences (Edgar et al., 2011). The true fungal OTUs in origin were used for taxonomic assignments ranging from phylum to species level based on the relative abundance of each OTU using Uparse ver. 7.0.1001 (Luo et al., 2012). OTUs identified as nonfungal were excluded from the analysis. A comparison between non-shared and shared OTUs was made based on a 97% similarity (Gao et al., 2019). A Venn diagram was generated to investigate differences in fungal communities among all soil samples, using the VennDiagram package (Chen and Boutros, 2011) in R statistical environment ver. 3.2.1 (R Core Team, 2020). Taxonomic assignment from the phylum to species levels was performed using UNITE 97% OTUs ITS reference sequences (Abarenkov et al., 2010). Classification of rarefied OTUs into an ecological guild was performed using the FUNGuild analysis pipeline (Nguyen et al., 2016), which enabled the detection of fungal functional groups based on the relative frequency of each OTU among respective sampling sites. Also, function cluster analysis based on the FUNGuild data was preceded by principal component analysis (PCA). Soil mycobiome diversity within samples (alpha diversity) and between samples (beta diversity) was assessed using QIIIME. The following seven indices, which reflect the richness and diversity of each sample, were used to measure alpha diversity: observed species, Shannon diversity index (Shannon-Wiener index or Shannon-Weaver index) (Shannon, 2001), Chao1 species richness estimator (Chao, 1984), phylogenetic diversity (PD whole tree) (Faith, 1992), abundance coverage-based estimator (ACE) (Chao and Lee, 1992), Good's coverage (Good, 1953), and Simpson (Simpson, 1949). We investigated whether the sampling measure was satisfactory for apprehending the fungal observed species by structuring a species accumulation plot using the ‘specaccum’ function in the R package ‘vegan’ ver 2.3–4 (Oksanen et al., 2013). The sequencing capability of the microbial community structure was recorded by evaluating coverage values, higher than 99% for each sampling site, separately. Shannon and Simpson indices represent species diversity, whereas Chao1 and ACE indices measure species richness. All diversity measures were obtained using the alpha diversity QIIME script. Weighted- and unweighted UniFrac distances were chosen to calculate the dissimilarity coefficient between pairwise samples (Lozupone and Knight, 2005; Lozupone et al., 2011). UPGMA cluster analysis was performed using an average linkage based on weighted and unweighted pair groups to cluster the soil samples. Non-metric multidimensional scaling (NMDS) analysis was applied based on the Bray-Curtis distance at the OTUs level for exploring and visualizing the fungal community compositions. Linear discriminant analysis effect size (LEfSe) was used to quantitatively analyze biomarkers from the phylum to species level within different sampling groups (Segata et al., 2011). We examined general patterns of fungal richness and diversity in different ecological habitats by finding the correlations between fungal Shannon diversity and fungal richness (Chao1 index) with geographical coordinates using a non-parametric model, such as the generalized additive model (GAM) in R (the mgcv package). The test was performed using the Akaike information criterion (AIC) score. To investigate the impact of geographical coordinates on the fungal community structure, we performed GAM using Bray-Curtis dissimilarity matrices (calculated with the ‘vegdist’ function within the vegan package) based on fungal [OTUs](https://www.sciencedirect.com/topics/agricultural-and-biological-sciences/otus) and geographical coordinate matrices, which were calculated for all sampling locations, using their spatial information on longitude, latitude and elevation. Seven co-occurrence networks were built based on ITS sequences at the genus level with the relative abundance of > 0.1% for soil mycobiome from K, M, Y, B, F, G, and SH regions using data from all 98 sampling sites (Wang et al., 2022). The visualization of each individual network was performed using Gephi ver. 0.9.2 using Fruchterman-Reingold layout algorithm (Bastian et al., 2009). The dependencies and interactions of fungal taxa were inferred by identifying the number of positive (co-presence) and negative (mutual exclusion) correlations. We consider positive relationships (positive interaction as the symbol of intense relationships; stronger relationships or higher interactions) based on the study of wang et al. (2021). Also, betweenness centrality was estimated, which contains the number of least paths going through a given node as a representative for this node's location concerning other nodes (Ma et al., 2016). The normality of data and homogeneity of variance were tested using the Shapiro–Wilk test and Levene's test, respectively (OriginLab, Northampton, MA, USA). Statistically significant differences among different alpha diversity indices were established using the analysis of variance (ANOVA), followed by a Tukey-Kramer HSD Post-hoc using SAS JMP® ver. 13.2.0 (SAS Institute Inc., Cary, NC, USA, 1989-2019). *P* values of less than 0.05 were considered statistically significant. The merged approach of β-NTI and Bray-Curtis-based RaupCrick metrics (RC-Bray) was applied to quantify the fungal community assembly processes (Stegen et al., 2013; 2015). The phylogenetic signal was tested by evaluating the relationship between the phylogenetic distance of pairwise OTUs and their corresponding environmental differences using “mantelcorrelog” (Stegen et al., 2012), for which the “cophenetic” function in the “picante” package (R 3.14) was used to determine the phylogenetic distances. The values of βNTI > +2 or < -2 designate a community dominated by deterministic processes in the form of homogenous selection and variable selection (Stegen et al., 2012). In contrast, when -2 < βNTI < +2, it shows that stochastic processes are driving in the community in the form of dispersal limitation, homogenous dispersal, and an undominated process (Stegen et al., 2015). In the present study, the fungal OTUs with relatively higher abundances (> 0.0001%) were selected (11,593 OTUs) to determine the β-NTI and RCbray values, according to Shi et al. (2020).

**Supplementary Figures**


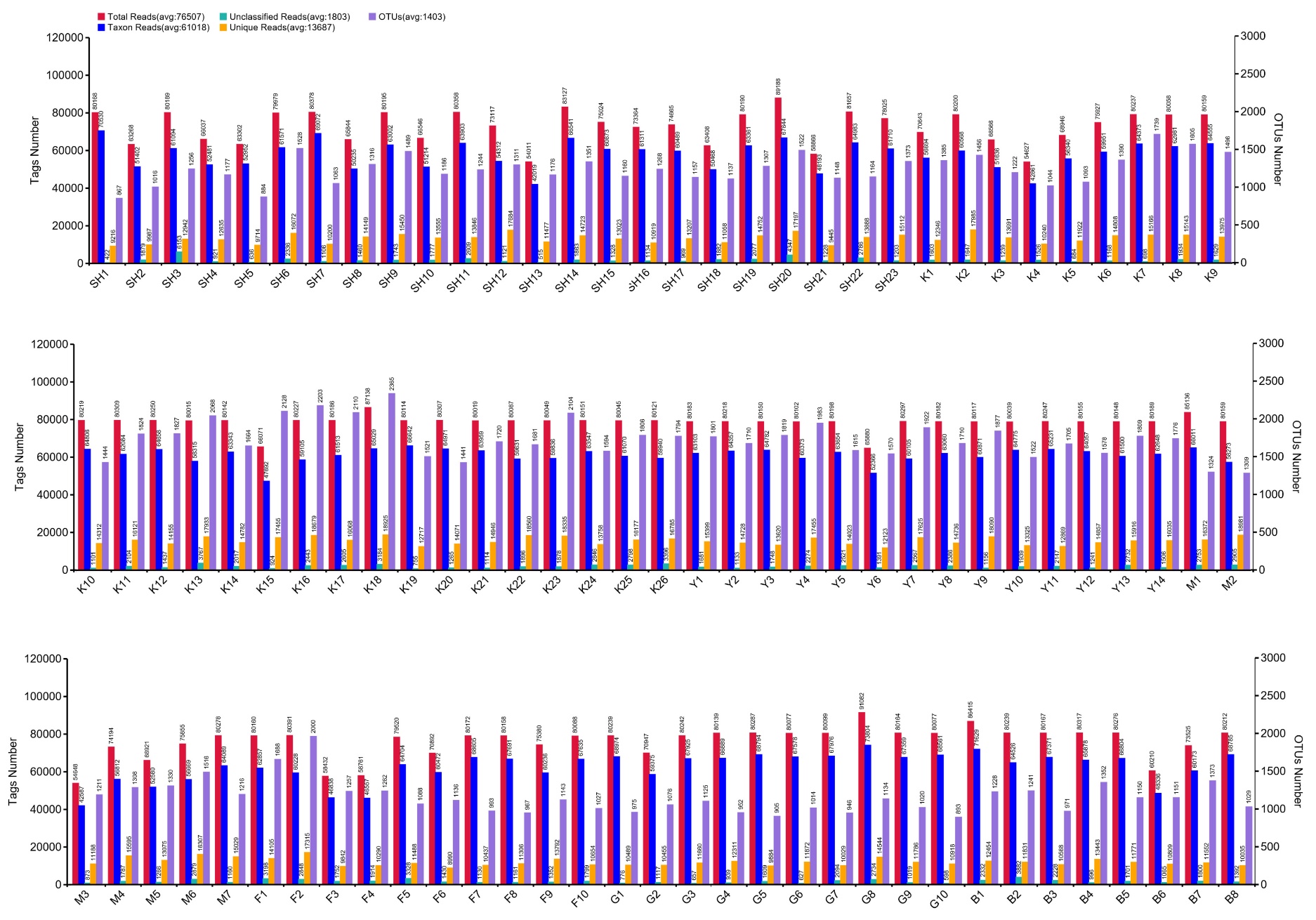


**Supplementary Figure S1**. Statistical analysis of the tags and OTU numbers for 98 soil samples.


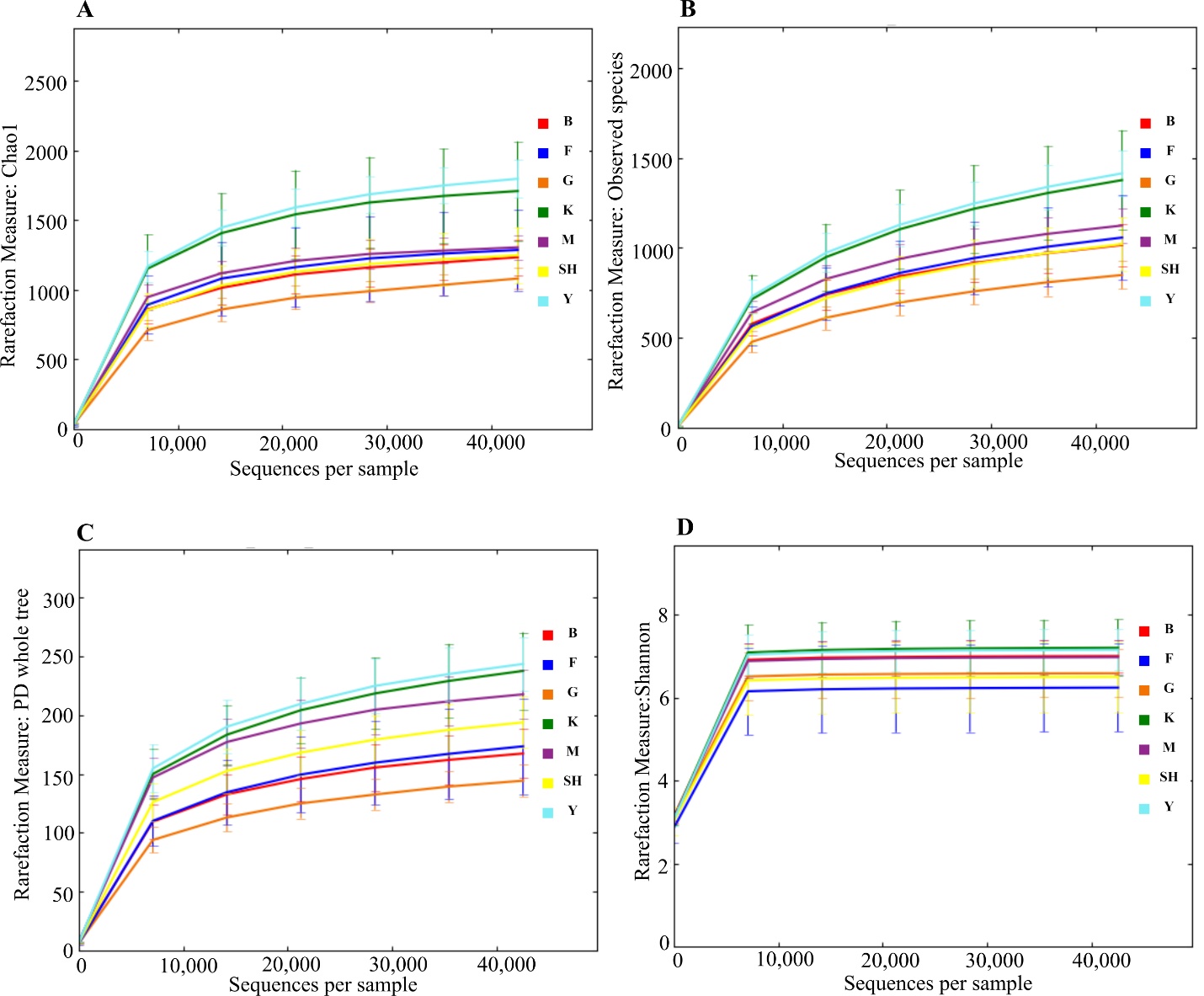


**Supplementary Figure S2**. Rarefaction curves representing the relation between the number of sequences and Chao1 estimator (A), observed species (B), PD whole tree (C), and Shannon index (D).


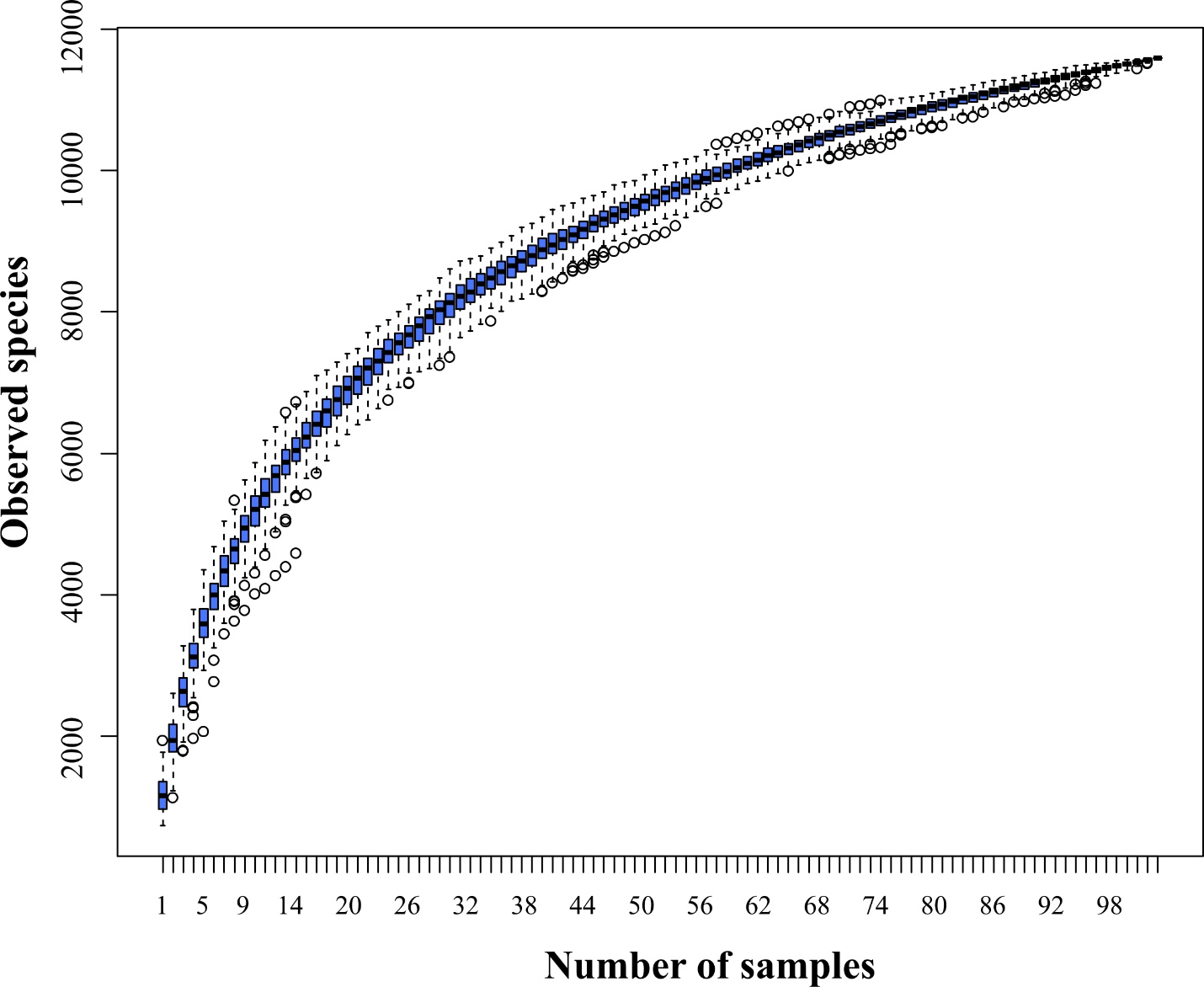


**Supplementary Figure S3**: Species accumulation curves of estimated observed species for soil fungal communities at an increasing number of samples. Solid line refers to the average estimated fungal observed species.


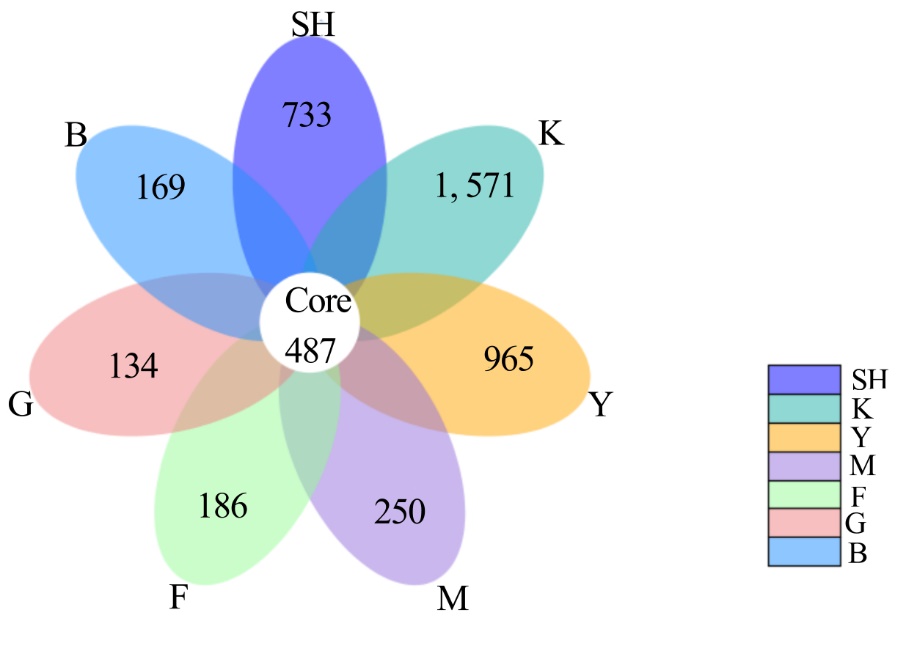


**Supplementary Figure S4**. Venn diagram showing the distribution of the unique and core fungal OTUs among 7 sampling locations with 98 sampling sites. Each petal in the flower chart represents a sampling site with different color. The core number in the middle represents the number of OTUs common to all sampling sites, and the number on the petals represents the unique OTUs for each sampling site.


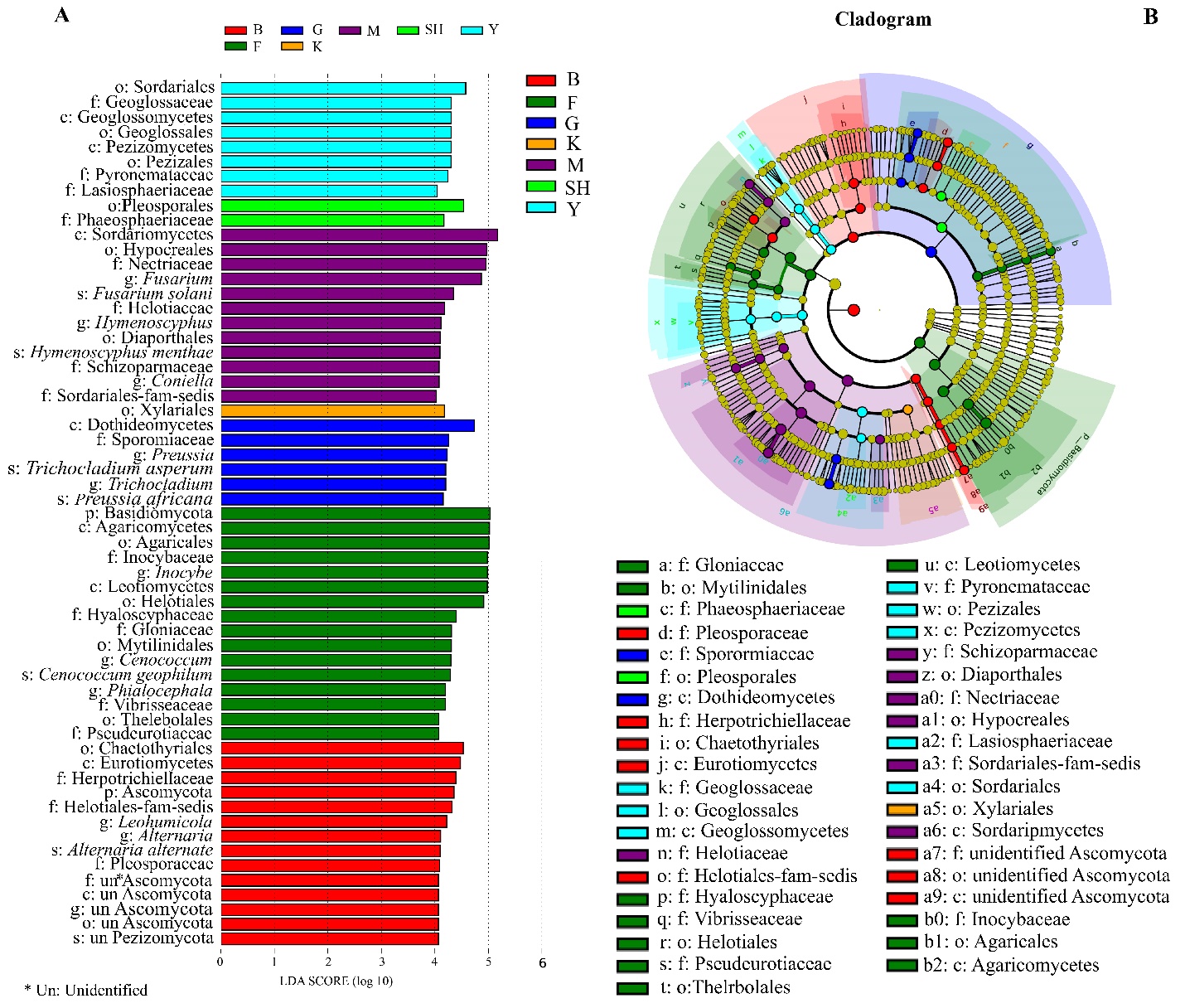


**Supplementary Figure S5**: Taxonomic differences among all sampling locations (A) Linear discriminative analysis (LDA) effective size (LEfSe) analysis. (B) Cladogram showing differentially abundant taxonomic clades with an LDA score > 4.0. Abbreviations: B indicates boundaries of forests and grasslands at SNFP; G, grasslands at SNFP; F, the forests at SNPF; SH, the Taihang Mountains; K, Meishan to Ping Ke Xiang; Y indicates Ya'an area; and M, Meishan region.


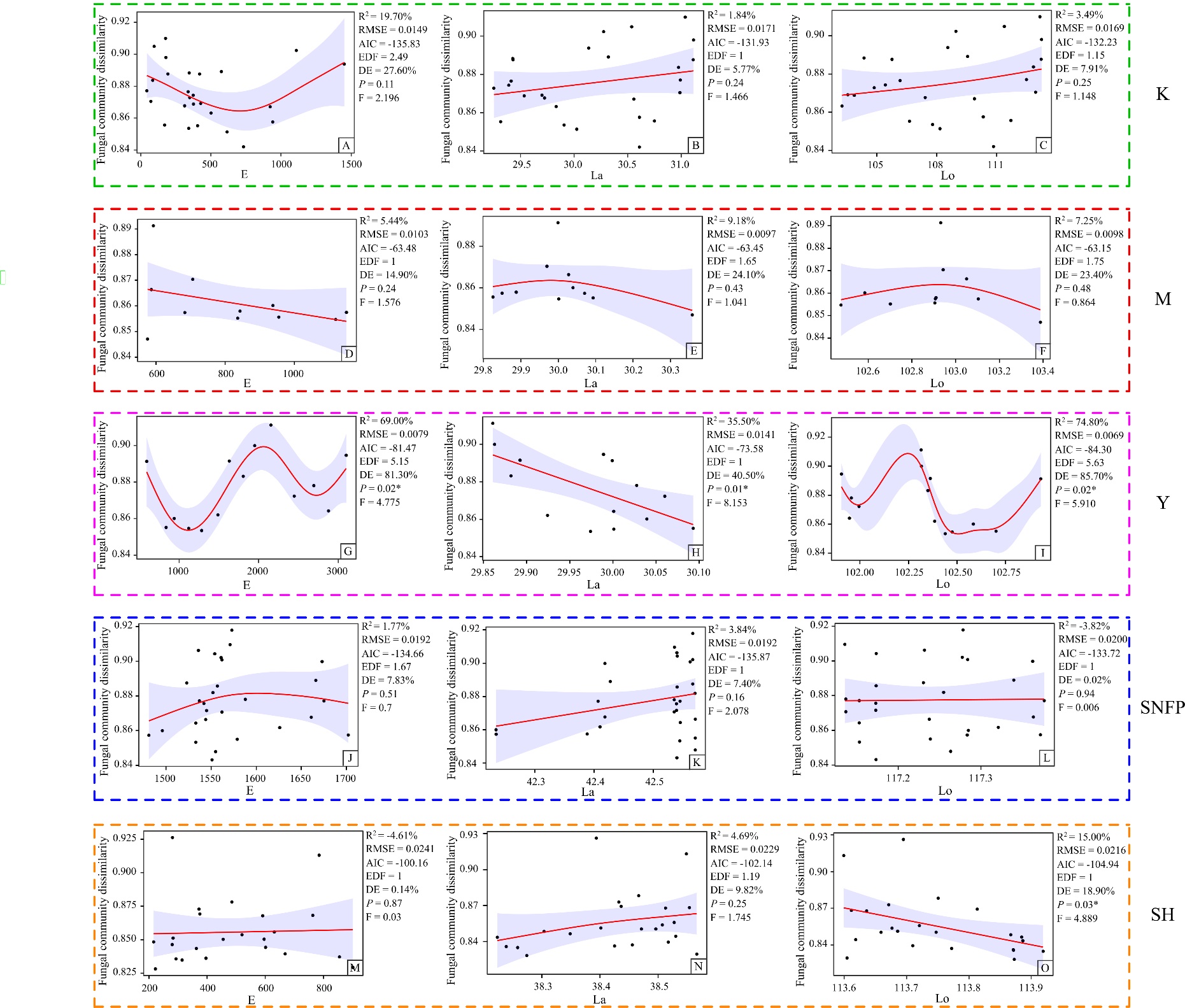


**Supplementary Figure S6**: Plots of fungal community dissimilarity vs. geographical coordinates. The Bray–Curtis dissimilarity measure was used to generate community distance matrices. The relationship between community dissimilarity and geographical coordinates was assessed by the use of a generalized additive model (GAM) for each data set. Abbreviations: K: Meishan to Ping Ke Xian, SH: Taihang Mountains, M: Meishan region, Y: Ya'an area, and SNFP: Saihanba National Forest Park.


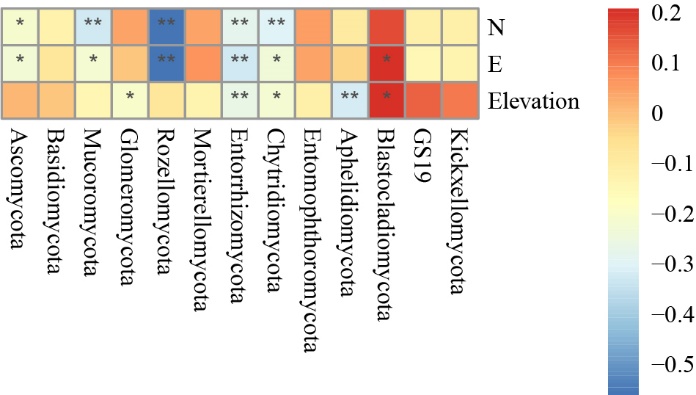


**Supplementary Figure S7**: Spearman correlation analysis of the relative abundance of dominant phyla with the GPS coordinates.


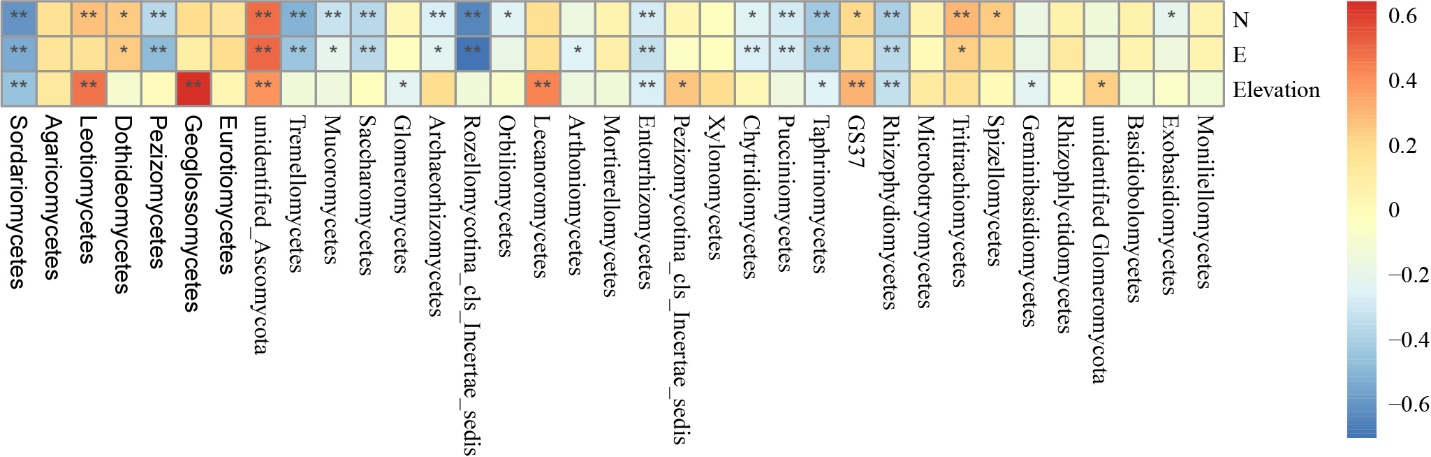


**Supplementary Figure S8**: Spearman correlation analysis of the relative abundance of dominant classes with the GPS coordinates.


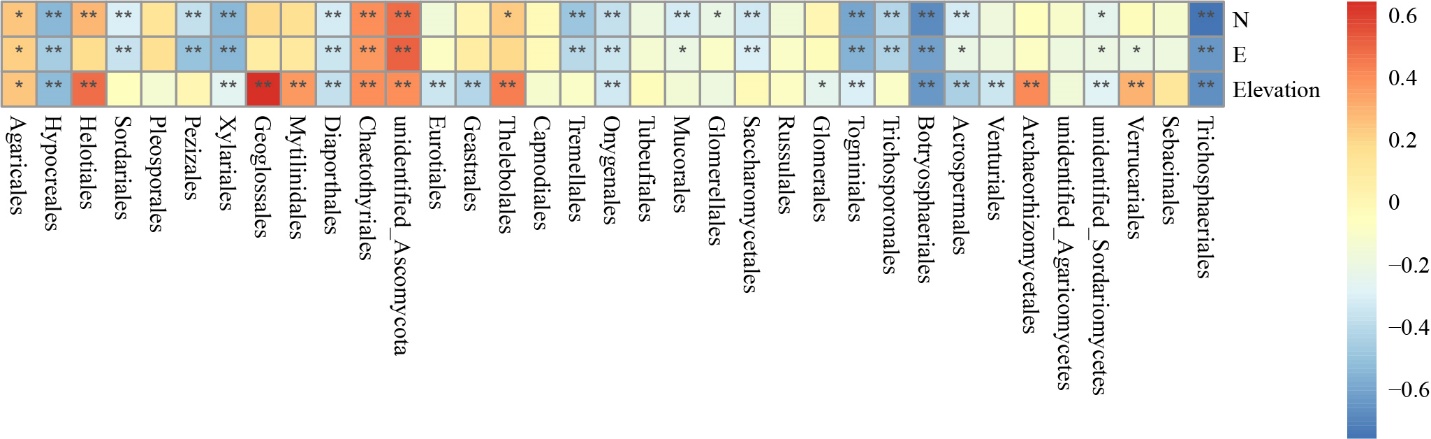


**Supplementary Figure S9**: Spearman correlation analysis of the relative abundance of dominant fungal orders with the GPS coordinates.


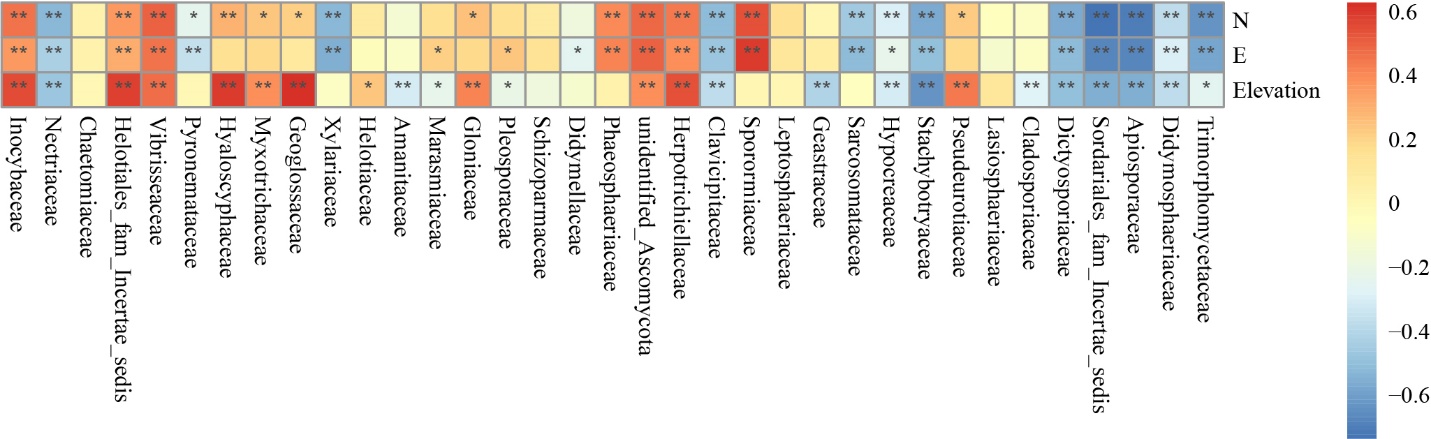


**Supplementary Figure S10**: Spearman correlation analysis of the relative abundance of dominant fungal families with the GPS coordinates.


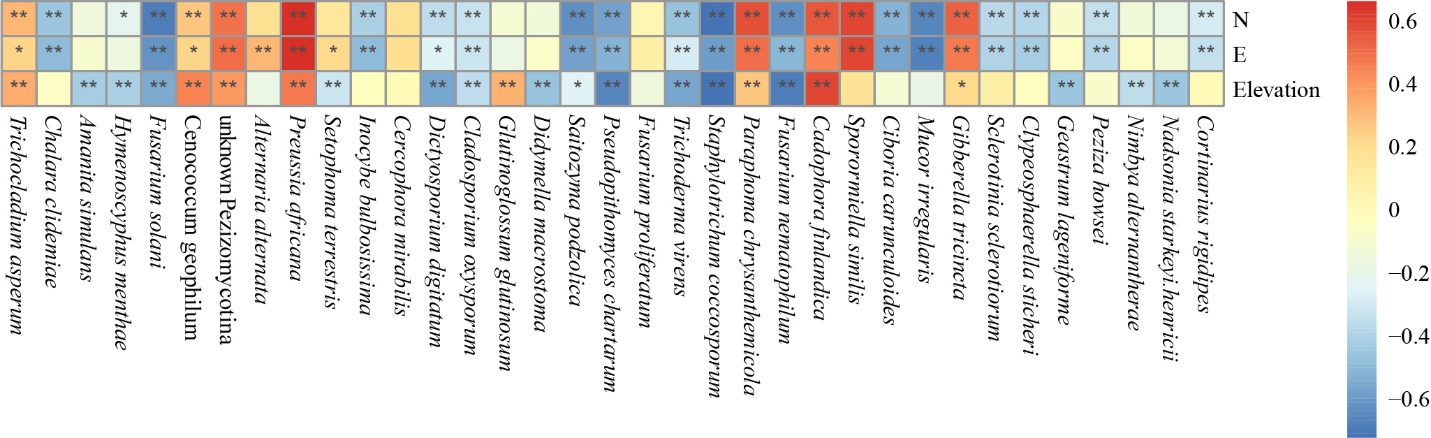


**Supplementary Figure S11**: Spearman correlation analysis of the relative abundance of dominant fungal species with the GPS coordinates.

**Supplementary Figure S12**:
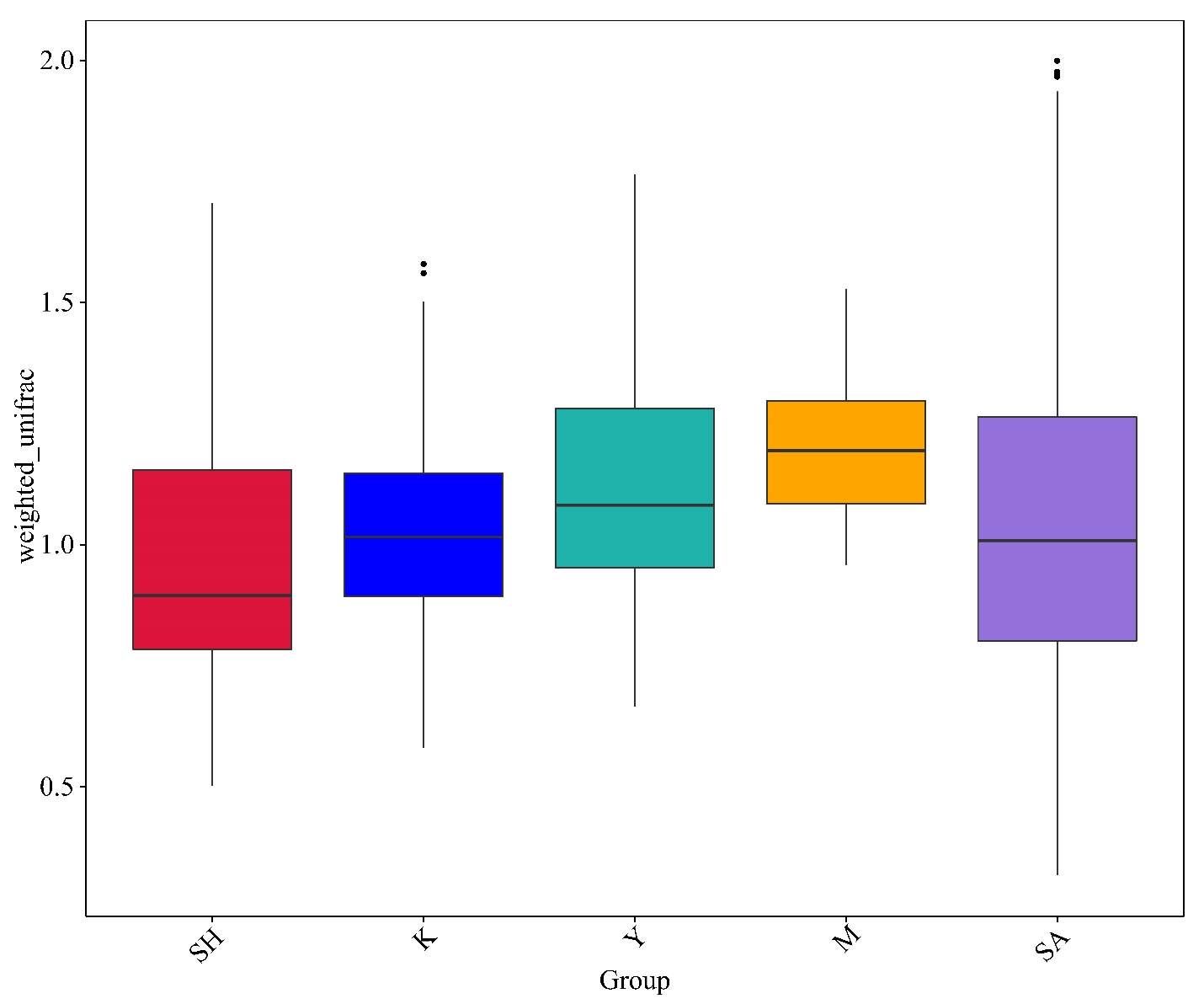
 Weighted UniFrac distance to measure fungal community compositions among sampling locations.

**Supplementary Tables**

| **Supplementary Table S1**: Sampling locations and GPS coordinates | | | |
| --- | --- | --- | --- |
| Locations | Latitude and Longitude | Elevation (m) |  |
| SH1 | 38° 23ʹ.618ʹʹ N, 113° 41ʹ.698ʹʹ E | 281 |  |
| SH2 | 38° 24ʹ.085ʹʹ N, 113° 41ʹ.144ʹʹ E | 283 |  |
| SH3 | 38° 25ʹ.938ʹʹ N, 113° 40ʹ.281ʹʹ E | 372 |  |
| SH4 | 38° 28ʹ.268ʹʹ N, 113° 39ʹ.538ʹʹ E | 455 |  |
| SH5 | 38° 30ʹ.757ʹʹ N, 113° 38ʹ.158ʹʹ E | 591 |  |
| SH6 | 38° 31ʹ.809ʹʹ N, 113° 37ʹ.105ʹʹ E | 601 |  |
| SH7 | 38° 32ʹ.906ʹʹ N, 113° 35ʹ.952ʹʹ E | 785 |  |
| SH8 | 38° 33ʹ.226ʹʹ N, 113° 36ʹ.690ʹʹ E | 763 |  |
| SH9 | 38° 33ʹ.981ʹʹ N, 113° 36ʹ. 265ʹʹ E | 901 |  |
| SH10 | 38° 30ʹ.433ʹʹ N, 113° 40ʹ.601ʹʹ E | 520 |  |
| SH11 | 38° 31ʹ.046ʹʹ N, 113° 42ʹ.596ʹʹ E | 668 |  |
| SH12 | 38° 31ʹ.647ʹʹ N, 113° 43ʹ.237ʹʹ E | 631 |  |
| SH13 | 38° 29ʹ.758ʹʹ N, 113° 44ʹ.849ʹʹ E | 596 |  |
| SH14 | 38° 27ʹ.988ʹʹ N, 113° 45ʹ.050ʹʹ E | 485 |  |
| SH15 | 38° 27ʹ.341ʹʹ N, 113° 46ʹ.343ʹʹ E | 855 |  |
| SH16 | 38° 26ʹ.183ʹʹ N, 113° 48ʹ.802ʹʹ E | 375 |  |
| SH17 | 38° 25ʹ.493ʹʹ N, 113° 52ʹ.267ʹʹ E | 396 |  |
| SH18 | 38° 20ʹ.924ʹʹ N, 113° 53ʹ.078ʹʹ E | 280 |  |
| SH19 | 38° 18ʹ.227ʹʹ N, 113° 52ʹ.036ʹʹ E | 216 |  |
| SH20 | 38° 16ʹ.444ʹʹ N, 113° 52ʹ.367ʹʹ E | 222 |  |
| SH21 | 38° 14ʹ.327ʹʹ N, 113° 52ʹ.296ʹʹ E | 293 |  |
| SH22 | 38° 13ʹ.406ʹʹ N, 113° 53ʹ.192ʹʹ E | 363 |  |
| SH23 | 38° 15ʹ.501ʹʹ N, 113° 55ʹ.149ʹʹ E | 314 |  |
| K1 | 29° 49ʹ.903ʹʹ N, 103° 14ʹ.656ʹʹ E | 501 |  |
| K2 | 29° 41ʹ.924ʹʹ N, 103° 32ʹ.266ʹʹ E | 427 |  |
| K3 | 29° 31ʹ.942ʹʹ N, 103° 51ʹ.702ʹʹ E | 375 |  |
| K4 | 29° 25ʹ.548ʹʹ N, 104° 20ʹ.811ʹʹ E | 345 |  |
| K5 | 29° 14ʹ.964ʹʹ N, 104° 51ʹ.560ʹʹ E | 348 |  |
| K6 | 29° 23ʹ.329ʹʹ N, 105° 25ʹ.040ʹʹ E | 373 |  |
| K7 | 29° 25ʹ.708ʹʹ N, 105° 45ʹ.298ʹʹ E | 419 |  |
| K8 | 29° 24ʹ.678ʹʹ N, 106° 09ʹ.517ʹʹ E | 337 |  |
| K9 | 29° 18ʹ.819ʹʹ N, 106° 37ʹ.694ʹʹ E | 404 |  |
| K10 | 29° 43ʹ.444ʹʹ N, 107° 25ʹ.003ʹʹ E | 312 |  |
| K11 | 29° 54ʹ.380ʹʹ N, 107° 47ʹ.463ʹʹ E | 341 |  |
| K12 | 30° 01ʹ.437ʹʹ N, 108° 09ʹ.753ʹʹ E | 615 |  |
| K13 | 30° 08ʹ.169ʹʹ N, 108° 32ʹ.994ʹʹ E | 1,451 |  |
| K14 | 30° 16ʹ.496ʹʹ N, 108° 57ʹ.363ʹʹ E | 1,109 |  |
| K15 | 30° 19ʹ.184ʹʹ N, 109° 32ʹ.665ʹʹ E | 574 |  |

| Continued |  |  |
| --- | --- | --- |
| Locations | Latitude and Longitude | Elevation (m) |
| K16 | 30° 33ʹ.465ʹʹ N, 109° 53ʹ.353ʹʹ E | 922 |
| K17 | 30° 36ʹ.596ʹʹ N, 110° 19ʹ.716ʹʹ E | 940 |
| K18 | 30° 36ʹ.595ʹʹ N, 110° 50ʹ.867ʹʹ E | 732 |
| K19 | 30° 32ʹ.264ʹʹ N, 111° 23ʹ.126ʹʹ E | 95 |
| K20 | 30° 45ʹ.015ʹʹ N, 111° 42ʹ.975ʹʹ E | 170 |
| K21 | 30° 59ʹ.550ʹʹ N, 112° 29ʹ.897ʹʹ E | 43 |
| K22 | 30° 58ʹ.525ʹʹ N, 112° 50ʹ.898ʹʹ E | 85 |
| K23 | 30° 59ʹ.322ʹʹ N, 112° 57ʹ.345ʹʹ E | 71 |
| K24 | 31° 02ʹ.297ʹʹ N, 113° 10ʹ.146ʹʹ E | 176 |
| K25 | 31° 06ʹ.732ʹʹ N, 113° 14ʹ.235ʹʹ E | 193 |
| K26 | 31° 06ʹ.962ʹʹ N, 113° 15ʹ.18ʹʹ E | 179 |
| Y1 | 29° 59ʹ.958ʹʹ N, 102° 55ʹ.899ʹʹ E | 592 |
| Y2 | 30° 05ʹ.590ʹʹ N, 102° 42ʹ.069ʹʹ E | 836 |
| Y3 | 30° 02ʹ.370ʹʹ N, 102° 35ʹ.042ʹʹ E | 938 |
| Y4 | 30° 00ʹ.069ʹʹ N, 102° 28ʹ.522ʹʹ E | 1,120 |
| Y5 | 29° 58ʹ.447ʹʹ N, 102° 26ʹ.355ʹʹ E | 1,283 |
| Y6 | 29° 55ʹ.457ʹʹ N, 102° 23ʹ.108ʹʹ E | 1,488 |
| Y7 | 29° 53ʹ.548ʹʹ N, 102° 21ʹ.753ʹʹ E | 1,631 |
| Y8 | 29° 52ʹ.917ʹʹ N, 102° 20ʹ.994ʹʹ E | 1,806 |
| Y9 | 29° 51ʹ.778ʹʹ N, 102° 18ʹ.983ʹʹ E | 1,950 |
| Y10 | 29° 51ʹ.652ʹʹ N, 102° 18ʹ.928ʹʹ E | 2,153 |
| Y11 | 30° 03ʹ.610ʹʹ N, 101° 59ʹ.712ʹʹ E | 2,447 |
| Y12 | 30° 01ʹ.662ʹʹ N, 101° 57ʹ.356ʹʹ E | 2,693 |
| Y13 | 30° 00ʹ.059ʹʹ N, 101° 56ʹ.750ʹʹ E | 2,877 |
| Y14 | 29° 59ʹ.354ʹʹ N, 101° 54ʹ.264ʹʹ E | 3,100 |
| M1 | 30° 01ʹ.655ʹʹ N, 103° 03ʹ.006ʹʹ E | 586 |
| M2 | 30° 04ʹ.212ʹʹ N, 103° 06ʹ.213ʹʹ E | 683 |
| M3 | 30° 21ʹ.498ʹʹ N, 103° 23ʹ.239ʹʹ E | 575 |
| M4 | 29° 50ʹ.985ʹʹ N, 102° 54ʹ.414ʹʹ E | 1,151 |
| M5 | 29° 53ʹ.255ʹʹ N, 102° 54ʹ.608ʹʹ E | 843 |
| M6 | 29° 58ʹ.183ʹʹ N, 102° 56ʹ.560ʹʹ E | 706 |
| M7 | 29° 49ʹ.516ʹʹ N, 102° 54ʹ.276ʹʹ E | 955 |
| F1 | 42° 32ʹ.375ʹʹ N, 117° 13ʹ.898ʹʹ E | 1,536 |
| F2 | 42° 33ʹ.975ʹʹ N, 117° 13ʹ.824ʹʹ E | 1,523 |
| F3 | 42° 34ʹ.249ʹʹ N, 117° 14ʹ.251ʹʹ E | 1,544 |
| F4 | 42° 34ʹ.236ʹʹ N, 117° 14ʹ.332ʹʹ E | 1,579 |
| F5 | 42° 34ʹ.238ʹʹ N, 117° 15ʹ.805ʹʹ E | 1,555 |
| F6 | 42° 33ʹ.973ʹʹ N, 117° 16ʹ.647ʹʹ E | 1,561 |

| Continued | | |
| --- | --- | --- |
| Locations | Latitude and Longitude | Elevation (m) |
| F7 | 42° 33ʹ.988ʹʹ N, 117° 16ʹ.701ʹʹ E | 1,573 |
| F8 | 42° 33ʹ.715ʹʹ N, 117° 17ʹ.041ʹʹ E | 1,562 |
| F9 | 42° 34ʹ.219ʹʹ N, 117° 15ʹ.294ʹʹ E | 1,552 |
| F10 | 42° 32ʹ.331ʹʹ N, 117° 10ʹ.370ʹʹ E | 1,542 |
| G1 | 42° 32ʹ.347ʹʹ N, 117° 10ʹ.368ʹʹ E | 1,545 |
| G2 | 42° 32ʹ.376ʹʹ N, 117° 10ʹ.386ʹʹ E | 1,551 |
| G3 | 42° 32ʹ.402ʹʹ N, 117° 10ʹ.394ʹʹ E | 1,557 |
| G4 | 42° 32ʹ.425ʹʹ N, 117° 10ʹ.401ʹʹ E | 1,554 |
| G5 | 42° 32ʹ.674ʹʹ N, 117° 09ʹ.182ʹʹ E | 1,533 |
| G6 | 42° 32ʹ.699ʹʹ N, 117° 09ʹ.173ʹʹ E | 1,537 |
| G7 | 42° 32ʹ.717ʹʹ N, 117° 09ʹ.169ʹʹ E | 1,533 |
| G8 | 42° 32ʹ.097ʹʹ N, 117° 08ʹ.199ʹʹ E | 1,562 |
| G9 | 42° 32ʹ.109ʹʹ N, 117° 08ʹ.193ʹʹ E | 1,588 |
| G10 | 42° 32ʹ.129ʹʹ N, 117° 08ʹ.124ʹʹ E | 1,571 |
| B1 | 42° 14ʹ.084ʹʹ N, 117° 17ʹ.005ʹʹ E | 1,481 |
| B2 | 42° 14ʹ.065ʹʹ N, 117° 17ʹ.085ʹʹ E | 1,496 |
| B3 | 42° 24ʹ.590ʹʹ N, 117° 19ʹ.262ʹʹ E | 1,626 |
| B4 | 42° 25ʹ.641ʹʹ N, 117° 20ʹ.387ʹʹ E | 1,666 |
| B5 | 42° 25ʹ.088ʹʹ N, 117° 21ʹ.758ʹʹ E | 1,673 |
| B6 | 42° 25ʹ.093ʹʹ N, 117° 21ʹ.812ʹʹ E | 1,661 |
| B7 | 42° 24ʹ.405ʹʹ N, 117° 22ʹ.596ʹʹ E | 1,675 |
| B8 | 42° 23ʹ.297ʹʹ N, 117° 22ʹ.316ʹʹ E | 1,702 |

| **Supplementary Table S2**: showing sample size, number of valid reads, numbers of OTUs for each sampling location, and classified and unclassified fungal taxa | | | | | | | | | | | |
| --- | --- | --- | --- | --- | --- | --- | --- | --- | --- | --- | --- |
| SL^1^ | SS^2^ | AR^3^ | ON^4^ | Kingdom | Phylum | Class | Order | Family | Genus | Species | UT^5^ |
| SH | 23 | 73,517 | 1,221 | 58,659 | 35,928 | 33,804 | 31,191 | 26,047 | 23,526 | 11,243 | 1,804 |
| K | 26 | 77,415 | 1,681 | 60,234 | 38,693 | 37,204 | 35,270 | 29,415 | 26,113 | 13,032 | 1,833 |
| Y | 7 | 79,150 | 1,742 | 62,205 | 43,226 | 40,380 | 39,385 | 32,596 | 25,988 | 12,379 | 1,887 |
| M | 14 | 73,884 | 1,316 | 56,717 | 37,540 | 35,431 | 33,994 | 31,433 | 28,380 | 13,722 | 1,946 |
| F | 10 | 74,395 | 1,256 | 60,582 | 46,698 | 45,967 | 44,925 | 39,778 | 34,305 | 10,653 | 1,991 |
| G | 10 | 80,335 | 1,004 | 67,723 | 40,886 | 36,860 | 32,870 | 27,193 | 20,557 | 12,862 | 1,217 |
| B | 8 | 77,670 | 1,186 | 64,187 | 40,466 | 38,534 | 36,137 | 29,589 | 24,500 | 11,308 | 1,924 |
| 1: Sampling locations; 2: Sample size; 3: Average reads; 4: OTU numbers; 5: Unclassified Tags | | | | | | | | | | | |

| **Supplementary Table** **S4**: ADONIS analysis | | | | | | |
| --- | --- | --- | --- | --- | --- | --- |
| Vs_group | Df | Sums Of Sqs | Mean Sqs | F Model | R^2^ | Pr (>F) |
| SH-K | 1(47) | 1.6567(15.7252) | 1.65675(0.33458) | 4.9517 | 0.09531(0.90469) | 0.001 |
| SH-Y | 1(35) | 1.6062(11.3977) | 1.60623(0.32565) | 4.9324 | 0.12352(0.87648) | 0.001 |
| SH-G | 1(31) | 1.6618(9.3848) | 1.66176(0.30273) | 5.4892 | 0.15043(0.84957) | 0.001 |
| SH-B | 1(29) | 1.5222(8.8302) | 1.52219(0.30449) | 4.9992 | 0.14704(0.85296) | 0.001 |
| SH-M | 1(28) | 0.9406(8.5867) | 0.94060(0.30667) | 3.0672 | 0.09873(0.90127) | 0.001 |
| SH-F | 1(31) | 1.9474(9.5018) | 1.94742(0.30651) | 6.3535 | 0.17009(0.82991) | 0.001 |
| K-Y | 1(38) | 0.9624(13.6025) | 0.96236(0.35796) | 2.6884 | 0.06607(0.93393) | 0.001 |
| K-G | 1(34) | 1.9633(11.5896) | 1.96335(0.34087) | 5.7598 | 0.14486(0.85514) | 0.001 |
| K-B | 1(32) | 1.6693(11.0350) | 1.66929(0.34484) | 4.8407 | 0.1314(0.8686) | 0.001 |
| K-M | 1(31) | 0.5527(10.7915) | 0.55267(0.34811) | 1.5876 | 0.04872(0.95128) | 0.005 |
| K-F | 1(34) | 1.9067(11.7067) | 1.90668(0.34431) | 5.5376 | 0.14006(0.85994) | 0.001 |
| Y-G | 1(22) | 1.4197(7.2621) | 1.41969(0.33009) | 4.3009 | 0.16353(0.83647) | 0.001 |
| Y-B | 1(20) | 1.1449(6.7075) | 1.14495(0.33537) | 3.414 | 0.14581(0.85419) | 0.001 |
| Y-M | 1(19) | 0.6504(6.4640) | 0.65037(0.34021) | 1.9117 | 0.09142(0.90858) | 0.003 |
| Y-F | 1(22) | 1.1888(7.3791) | 1.18878(0.33541) | 3.5442 | 0.13875(0.86125) | 0.001 |
| G-B | 1(16) | 0.4322(4.6946) | 0.43216(0.29341) | 1.4729 | 0.0843(0.9157) | 0.053 |
| G-M | 1(15) | 1.4219(4.4511) | 1.42191(0.29674) | 4.7918 | 0.24211(0.75789) | 0.001 |
| G-F | 1(18) | 0.9988(5.3662) | 0.99879(0.29812) | 3.3502 | 0.15692(0.84308) | 0.001 |
| B-M | 1(13) | 1.2997(3.8965) | 1.29966(0.29973) | 4.3361 | 0.25012(0.74988) | 0.001 |
| B-F | 1(16) | 0.5420(4.8116) | 0.54200(0.30073) | 1.8023 | 0.10124(0.89876) | 0.034 |
| M-F | 1(15) | 1.4066(4.5681) | 1.40661(0.30454) | 4.6188 | 0.23543(0.76457) | 0.001 |

| **Supplementary Table** **S5**: MRPP statistical analysis using Spearman's rank correlation. | | | | |
| --- | --- | --- | --- | --- |
| Group | A | observed-delta | expected-delta | Significance |
| F-G | 0.0579 | 0.7659 | 0.813 | 0.002 |
| F-M | 0.09635 | 0.7749 | 0.8575 | 0.001 |
| G-M | 0.09819 | 0.7677 | 0.8512 | 0.001 |
| B-F | 0.02362 | 0.7694 | 0.788 | 0.038 |
| B-G | 0.01372 | 0.7626 | 0.7732 | 0.064 |
| B-M | 0.09885 | 0.7721 | 0.8568 | 0.001 |
| F-K | 0.06141 | 0.8247 | 0.8786 | 0.001 |
| G-K | 0.06353 | 0.8212 | 0.8769 | 0.001 |
| K-M | 0.01089 | 0.8309 | 0.8401 | 0.002 |
| B-K | 0.05521 | 0.8264 | 0.8746 | 0.001 |
| F-Y | 0.05379 | 0.8134 | 0.8597 | 0.001 |
| G-Y | 0.06609 | 0.8083 | 0.8655 | 0.001 |
| M-Y | 0.02313 | 0.8217 | 0.8411 | 0.002 |
| B-Y | 0.0548 | 0.815 | 0.8623 | 0.001 |
| K-Y | 0.02109 | 0.8442 | 0.8624 | 0.001 |
| F-SH | 0.07446 | 0.7775 | 0.8401 | 0.001 |
| G-SH | 0.06356 | 0.7738 | 0.8263 | 0.001 |
| M-SH | 0.03391 | 0.7797 | 0.8071 | 0.001 |
| B-SH | 0.06008 | 0.7764 | 0.826 | 0.001 |
| K-SH | 0.0399 | 0.8144 | 0.8482 | 0.001 |
| SH-Y | 0.04998 | 0.8038 | 0.8461 | 0.001 |

| **Supplementary Table S6**: top 10 nodes with highest betweenness centrality (BC) value for each sampling location | | |
| --- | --- | --- |
| Genera | BC | Locations |
| *Didymella* | 2 | K |
| *Dactylonectria* | 1 |  |
| *Setophoma* | 0 |  |
| *Fusarium* | 0 |  |
| *Gibberella* | 0 |  |
| *Trichocladium* | 0 |  |
| *Alternaria* | 0 |  |
| *Pseudopithomyces* | 0 |  |
| *Cladosporium* | 0 |  |
| *Cylindrocladiella* | 418.2455 | M |
| *Mucor* | 328.4392 |  |
| *Ustilaginoidea* | 327.0522 |  |
| *Diaporthe* | 258.8428 |  |
| *Aspergillus* | 246.1545 |  |
| *Didymella* | 240.9068 |  |
| *Beauveria* | 236.0581 |  |
| *Annulohypoxylon* | 217.48 |  |
| *Hymenoscyphus* | 214.4076 |  |
| *Penicillium* | 213.6685 |  |
| *Alternaria* | 518.483 | Y |
| *Capronia* | 310.7908 |  |
| *Inocybe* | 304.7808 |  |
| *Trichocladium* | 216.2555 |  |
| *Tetracladium* | 214.4499 |  |
| *Glomus* | 210.8264 |  |
| *Nigrospora* | 205.1003 |  |
| *Lasiodiplodia* | 178.5819 |  |
| *Staphylotrichum* | 167.5846 |  |
| *Epicoccum* | 164.2406 |  |
| *Fusarium* | 201.1146 | G |
| *Coniella* | 185.6632 |  |
| *Pyrenochaeta* | 168.2612 |  |
| *Gibberella* | 139.7238 |  |
| *Chaetomium* | 122.654 |  |
| *Fusicolla* | 106.7793 |  |
| *Cephalosporium* | 104 |  |
| *Leptodontidium* | 103.0767 |  |
| *Chalara* | 94.69678 |  |
| *Dactylospora* | 78.8653 |  |
| *Herpotrichia* | 572.8767 | B |
| *Muriphaeosphaeria* | 527.2732 |  |
| *Prosthemium* | 504 |  |
| *Capronia* | 338.9726 |  |
| *Apodus* | 300 |  |
| *Cercophora* | 277.5071 |  |
| *Trichoderma* | 246.8086 |  |
| *Titaea* | 244 |  |
| *Inocybe* | 238.7517 |  |
| *Trichophaea* | 233.9071 |  |
| *Cephalosporium* | 294.1863 | F |
| *Cortinarius* | 270.4564 |  |
| *Paraphoma* | 222.3537 |  |
| *Setophoma* | 194.4145 |  |
| *Ilyonectria* | 178.835 |  |
| *Capronia* | 175.8674 |  |
| *Neonectria* | 175.5322 |  |
| *Clonostachys* | 156 |  |
| *Lachnum* | 125.5773 |  |
| *Trichocladium* | 118.8726 |  |
| *Aspergillus* | 22 | SH |
| *Staphylotrichum* | 17.5 |  |
| *Pyrenochaeta* | 5 |  |
| *Tetracladium* | 3 |  |
| *Chaetomium* | 2.5 |  |
| *Fusarium* | 0.5 |  |
| *Pseudopithomyces* | 0.5 |  |
| *Trichocladium* | 0 |  |
| *Preussia* | 0 |  |
| *Setophoma* | 0 |  |

**References**

Abarenkov, K., Henrik Nilsson, R., Larsson, K.-H., Alexander, I.J., Eberhardt, U., Erland, S., Høiland, K., Kjøller, R., Larsson, E., Pennanen, T., Sen, R., Taylor, A.F.S., Tedersoo, L., Ursing, B.M., Vrålstad, T., Liimatainen, K., Peintner, U., and Kõljalg, U., (2010). The UNITE database for molecular identification of fungi-recent updates and future perspectives. New Phytol. 186 (2), 281-285. DOI: <https://doi.org/10.1111/j.1469-8137.2009.03160.x>.

Bastian, M., Heymann, S., and Jacomy, M., 2009. Gephi: an open source software for exploring and manipulating networks. Proceedings of the International AAAI Conference on Web and Social Media 3, 361–362.

Chao, A., (1984). Nonparametric estimation of the number of classes in a population. Scand. J. Stat. 11 (4), 265-270. DOI: <https://www.jstor.org/stable/4615964>.

Chao, A., and Lee, S.-M., (1992). Estimating the number of classes via sample coverage. J. Am. Stat. Assoc. 87 (417), 210-217. DOI: <https://doi.org/10.2307/2290471>.

Chen, H., and Boutros, P.C., (2011). VennDiagram: a package for the generation of highly-customizable Venn and Euler diagrams in R. BMC Bioinformatics 12 (1), 35. DOI: <https://doi.org/10.1186/1471-2105-12-35>.

Edgar, R.C., Haas, B.J., Clemente, J.C., Quince, C., and Knight, R., (2011). UCHIME improves sensitivity and speed of chimera detection. Bioinformatics 27 (16), 2194-2200. DOI: <https://doi.org/10.1093/bioinformatics/btr381>.

Faith, D.P., (1992). Conservation evaluation and phylogenetic diversity. Biol. Conserv. 61 (1), 1-10. DOI: <https://doi.org/10.1016/0006-3207(92)91201-3>.

Gao, W., Zheng, C., Lei, Y., and Kuang, W., (2019). Analysis of bacterial communities in white clover seeds via high-throughput sequencing of 16S rRNA gene. Curr. Microbiol. 76 (2), 187-193. DOI: <https://doi.org/10.1007/s00284-018-1607-9>.

Good, I.J., (1953). The population frequencies of species and the estimation of population parameters. Biometrika 40 (3-4), 237-264. DOI: <https://doi.org/10.2307/2333344>.

Lozupone, C., and Knight, R., (2005). UniFrac: a new phylogenetic method for comparing microbial communities. Appl. Environ. Microbiol. 71 (12), 8228-8235. DOI: <https://doi.org/10.1128/AEM.71.12.8228-8235.2005>.

Lozupone, C., Lladser, M.E., Knights, D., Stombaugh, J., and Knight, R., (2011). UniFrac: an effective distance metric for microbial community comparison. ISME J. 5 (2), 169-172. DOI: <https://doi.org/10.1038/ismej.2010.133>.

Luo, C., Tsementzi, D., Kyrpides, N., Read, T., and Konstantinidis, K.T., (2012). Direct comparisons of Illumina vs. Roche 454 sequencing technologies on the same microbial community DNA sample. PLoS One 7 (2), e30087. DOI: <https://doi.org/10.1371/journal.pone.0030087>.

Ma, B., Wang, H., Dsouza, M., Lou, J., He, Y., Dai, Z., Brookes, P.C., Xu, J., and Gilbert, J.A., (2016). Geographic patterns of co-occurrence network topological features for soil microbiota at continental scale in eastern China. ISME J. 10 (8), 1891-1901. DOI: <https://doi.org/10.1038/ismej.2015.261>.

Nguyen, N.H., Song, Z., Bates, S.T., Branco, S., Tedersoo, L., Menke, J., Schilling, J.S., and Kennedy, P.G., (2016). FUNGuild: An open annotation tool for parsing fungal community datasets by ecological guild. Fungal Ecol. 20, 241-248. DOI: <https://doi.org/10.1016/j.funeco.2015.06.006>.

Oksanen, J., Blanchet, F.G., Kindt, R., Legendre, P., Minchin, P.R., O’hara, R., Simpson, G.L., Solymos, P., Stevens, M.H.H., and Wagner, H., (2013). Package ‘vegan’. Community ecology package, version 2 (9), 1-295.

Segata, N., Izard, J., Waldron, L., Gevers, D., Miropolsky, L., Garrett, W.S., and Huttenhower, C., (2011). Metagenomic biomarker discovery and explanation. Genome Biol. 12 (6), 1-18. DOI: <https://doi.org/10.1186/gb-2011-12-6-r60>.

Shannon, C., (2001). A mathematical theory of communication. Mob. Comput. Commun. Rev. 5, 3–55. DOI: <https://doi.org/10.1145/584091.584093>.

Shi, Y., Dang, K., Dong, Y., Feng, M., Wang, B., Li, J., and Chu, H., (2020). Soil fungal community assembly processes under long-term fertilization. Eur. J. Soil Sci. 71 (4), 716-726. DOI: <https://doi.org/10.1111/ejss.12902>.

Simpson, E.H., (1949). Measurement of diversity. Nature 163 (4148), 688-688. DOI: <https://doi.org/10.1038/163688a0>.

Stegen, J.C., Lin, X., Fredrickson, J.K., Chen, X., Kennedy, D.W., Murray, C.J., Rockhold, M.L., and Konopka, A., (2013). Quantifying community assembly processes and identifying features that impose them. ISME J. 7 (11), 2069-2079. DOI: <https://doi.org/10.1038/ismej.2013.93>.

Stegen, J.C., Lin, X., Fredrickson, J.K., and Konopka, A.E., (2015). Estimating and mapping ecological processes influencing microbial community assembly. Front. Microbiol. 6 (370). DOI: <https://doi.org/10.3389/fmicb.2015.00370>.

Stegen, J.C., Lin, X., Konopka, A.E., and Fredrickson, J.K., (2012). Stochastic and deterministic assembly processes in subsurface microbial communities. ISME J. 6 (9), 1653-1664. DOI: <https://doi.org/10.1038/ismej.2012.22>.

Wang, M., Masoudi, A., Wang, C., Yang, J., Zhai, Y., Wu, C., Yu, Z., and Liu, J., (2022). Plantation type and afforestation age disclose variable influences on soil microbial compositions in man-made forests in the Xiong'an New Area, China. Land Degrad. Dev., 1-16. DOI: <https://doi.org/10.1002/ldr.4372>.
